# Parallel comparative proteomics and phosphoproteomics reveal that cattle *myostatin* regulates phosphorylation of key enzymes in glycogen metabolism and glycolysis pathway

**DOI:** 10.18632/oncotarget.24250

**Published:** 2018-01-13

**Authors:** Shuping Yang, Xin Li, Xinfeng Liu, Xiangbin Ding, Xiangbo Xin, Congfei Jin, Sheng Zhang, Guangpeng Li, Hong Guo

**Affiliations:** ^1^ College of Animal Science and Veterinary Medicine, Tianjin Agricultural University, Tianjin 300384, China; ^2^ The Key Laboratory of Mammalian Reproductive Biology and Biotechnology of the Ministry of Education, Inner Mongolia University, Hohhot 010070, China; ^3^ Institute of Biotechnology, Cornell University, Ithaca, NY 14853, U.S.A

**Keywords:** myostatin, proteome, phosphoproteome, glycolysis, glycogenolysis

## Abstract

*MSTN*-encoded myostatin is a negative regulator of skeletal muscle development. Here, we utilized the gluteus tissues from MSTN gene editing and wild type Luxi beef cattle which are native breed of cattle in China, performed tandem mass tag (TMT) -based comparative proteomics and phosphoproteomics analyses to investigate the regulatory mechanism of *MSTN* related to cellular metabolism and signaling pathway in muscle development. Out of 1,315 proteins, 69 differentially expressed proteins (DEPs) were found in global proteomics analysis. Meanwhile, 149 differentially changed phosphopeptides corresponding to 76 unique phosphorylated proteins (DEPPs) were detected from 2,600 identified phosphopeptides in 702 phosphorylated proteins. Bioinformatics analyses suggested that majority of DEPs and DEPPs were closely related to glycolysis, glycogenolysis, and muscle contractile fibre processes. The global discovery results were validated by Multiple Reaction Monitoring (MRM)-based targeted peptide quantitation analysis, western blotting, and muscle glycogen content measurement. Our data revealed that increase in abundance of key enzymes and phosphorylation on their regulatory sites appears responsible for the enhanced glycogenolysis and glycolysis in *MSTN*^*−/−*^. The elevated glycogenolysis was assocaited with an enhanced phosphorylation of Ser1018 in PHKA1, and Ser641/Ser645 in GYS1, which were regulated by upstream phosphorylated AKT-GSK3β pathway and highly consistent with the lower glycogen content in gluteus of *MSTN*^*−/−*^. Collectively, this study provides new insights into the regulatory mechanisms of *MSTN* involved in energy metabolism and muscle growth.

## INTRODUCTION

*Myostatin* (growth and differentiation factor-8, GDF-8, encoded by *MSTN*), a member of the transforming growth factor-β (TGF-β) super family, is a critical autocrine/paracrine negative regulator of skeletal muscle mass during embryogenesis and early postnatal muscle growth [1, 2]. It is predominantly synthesized and expressed in skeletal muscle and shows a high degree of homology in gene structure and biological activity across a broad spectrum of animals [3]. Both *in vitro* and *in vivo* studies showed that myostatin signaling is mediated by binding to the activin type IIB receptor (ActRIIB). Dysfunction of myostatin by *MSTN* gene disruption, including natural gene mutation or gene modification or artificial myostatin protein inhibition, commonly results in ‘double-muscling’ phenotype which primarily shows a significant increase in muscle mass. In this phenotype, either the fibre number is increased (hyperplasia) and/or the individual muscle fibre size is raised (hypertrophy) [3–6] in many animals such as mice, sheep, dogs, cattle and humans [3, 5, 7–12], underscoring the well-conserved biological function of *MSTN* in muscle. In contrast, increased expression of *MSTN* usually leads to a reduction of muscle mass [13]. Potential mechanisms for *MSTN*’s inhibitory effect during myogenesis have been investigated in many studies, indicating that through a TGF-β [14, 15] pathway, *MSTN* can regulate many signaling cascades such as Wnt4/β-catenin signal pathway [16], IGF-I pathway [17], MAPK pathway [18] and PI3K/Akt pathway [17, 19]. Moreover, *MSTN* can also cause G1 arrest of myoblasts in cell cycle [20] and inhibit DNA and protein synthesis in myotubes, thus affecting myocyte proliferation [21]. Besides its effect on muscle development, *MSTN* plays a vital role in fat and glucose metabolism. Several studies show that *MSTN* not only inhibits the proliferation and differentiation of fat cells [22, 23] reducing adipogenesis [24, 25], but also decreases the expression of glucose transporter-4 (*GLUT4*) in bovine skeletal muscles [26] and insulin-stimulated glucose uptake in mouse liver cells [27]. Furthermore, knockout of the *MSTN* gene resulted in an increased abundance of some glycolytic proteins [28]. Due to the broad impact of *MSTN* on myogenesis, fat metabolism and glucose metabolism, high levels of myostatin have been shown to lead to muscle wasting that is associated with a variety of diseases, such as cancer [29], HIV infection [30], liver disease [31], obesity, insulin resistance and type II diabetes [24, 32, 33]. Therefore, pharmacological blockage of the myostatin/ActRIIB pathway is being actively pursued as a potential strategy for the treatment of muscle wasting diseases, and obesity [34–36]. Despite the central role of myostatin signaling cascades in regulation of muscle weight, the mechanism of this signaling cascade and action are still not fully understood [37]. Comprehensive analysis of *MSTN*’s regulation and signaling mechanism is also important in translational medicine field as it would shed light on the diagnosis and treatment of related diseases, animal production and food nutrition.

Since the wide application of omics technologies for system biology, considerable progress has been made in understanding protein functions and their cellular networks through qualitative and quantitative analyses of global proteome and its interaction complexes in response to specific perturbation [38, 39]. Many *MSTN* related protein studies have been carried out by a variety of methods [3, 16, 28, 40, 41]. However, most of the previous studies focused on the transgenic animal model of *MSTN*-null mice or natural double-muscled animals and on a single or a small number of targeted proteins. Very little research on heavy livestock with *MSTN* genetically engineered manipulation for global analysis of *MSTN* related proteome has been conducted and reported due to the long cultivation cycle, high-cost and other limitations. Moreover, protein phosphorylation affecting various processes such as cell growth, proliferation, survival, migration and metabolism. Signals governing these processes can be transduced through cascades of protein phosphorylation and dephosphorylation, enzymatically governed by protein kinases and protein phosphatases. However, no global phosphoproteomics analysis of the *MSTN* associated effect has been reported and therefore, the potential *MSTN* regulatory mechanisms through protein phosphorylation in their signaling cascades remain unknown.

In this study, to investigate the regulation mechanism of *MSTN* related to metabolism or signaling pathway in muscle development, we applied a 6-plex TMT labeling approach and conducted simultaneous measurements of global protein abundance and the phosphorylation status of cattle gluteus muscle proteins between *MSTN*^*−/−*^ cattle and WT cattle. These parallel proteomics and phosphoproteomics analyses enable us to compare changes in abundance of the phosphorylation state of a given protein to those in the overall abundance of that protein. After statistical and bioinformatics analyses and based on the results of changed abundance of proteins and phosphorylated sites/peptides relevant to the *MSTN* gene disruption, we carried out validation experiments including MRM-based peptide quantitation, Western blotting, enzyme-linked immuno sorbent assay for 16 DEPs/DEPPs as well as measurement of glycogen content and confirmed that the data sets from global discovery proteomics are reliable.

## RESULTS

### Identification of differentially expressed proteins (DEPs) between *MSTN*^*−/−*^ and WT

**Figure 1:**
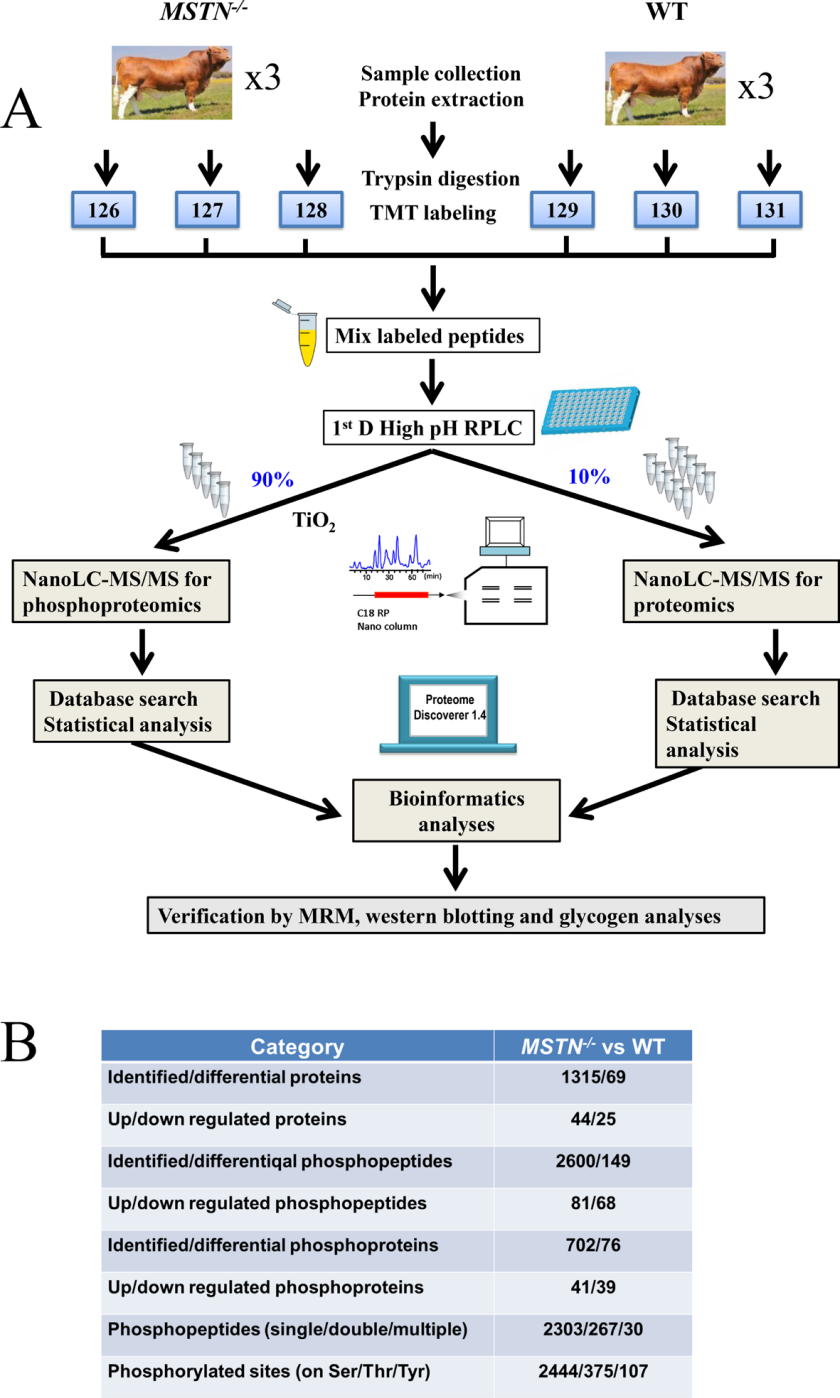
Experimental design and summary results using TMT 6-plex strategy (**A**) Experimental design and schematic diagram of the workflow. Luxi beef cattle was chosen for analysis of the differential proteomes between the *MSTN*^*−/−*^ and the WT beef cattle. A total of 6 cattle were analyzed by TMT 6-plex based shotgun-based parallel quantitative proteomics and phosphoproteomics, using the hpRP-nanoLC-MS/MS workflow. After thorough statistical analyses of the proteomics data, the differentially expressed proteins and phosphoproteins/sites were conducted for the subsequent bioinformatics analysis and some of the important differentially expressed proteins were selected for validation of the discovery results by multiple methods including sequence alignment, MRM, western blotting and ELISA approaches. (**B**) Summary of proteomics and phosphoproteomics results.

*MSTN*^*−/−*^ associated changes in abundance of any identified proteins were determined based on the TMT 6-plex reporter ion ratios. A total of 1,315 quantified proteins in gluteus muscle tissues of Luxi beef cattle containing at least two unique peptides per protein were identified in the data set (Supplementary Table 1A). After scatter plotting analysis used for determining the internal error of the biological replicates and student *t*-test analysis of the data set, the fold change in values more than 1.3 were determined based on the value of the log_2_ TMT ratio (log_2_ 1.3 = 0.38) at which 95% of all proteins had no deviation [42]. Thus, the fold-change (≥ 1.3) and *p*-value (≤ 0.05) from *t*-test were applied to rank and filter the quantitative data. The proteins with fold change ≥ 1.30 or ≤ 0.77 in relative abundance and a *p*-value ≤ 0.05 were identified as DEPs (Supplementary Table 1A). As a result, 69 DEPs containing 44 proteins in increased abundance and 25 in decreased abundance (Figure 1B) were identified in *MSTN*^−/−^ and used for subsequent bioinformatics analysis and selected validation experiments. The summary of the 69 DEPs by functional categories are listed in Supplementary Table 1B.

GO analysis shows that the functions of the 44 up-regulated proteins were classified into the categories of glycometabolism related proteins (34%), muscle related proteins (20%), fat metabolism related proteins (11%). While the 25 down-regulated proteins were classified to the categories of glycometabolism related proteins (8%), muscle related proteins (56%), fat metabolism related proteins (8%) (Supplementary Figures 1A, 1B). Interestingly, we found that almost all glycolytic proteins including some key enzymes in the glycolysis pathway (such as phosphoglucomutase-1, glucose-6-phosphate isomerase and pyruvate kinase), had an increased abundance in *MSTN*^*−/−*^ (Table 1). This result is expected to promote glycolytic activity and therefore provide more energy for muscle contraction and growth in *MSTN*^*−/−*^. Furthermore, our results show that slow twitch fibre related proteins (e.g. myosin-7 and myosin-2) were decreased in abundance, whereas fast twitch fibre related proteins (e.g. myosin-1 and troponin T, fast skeletal muscle isoform X32) were significantly up-regulated (Table 1). Notably, the increased abundance of both fast twitch fibre proteins and glycolysis proteins in *MSTN*^*−/−*^ would be selectively beneficial to the development and metabolism of fast muscle. And these results are consistent with the observed double muscle phenotype.

**Table 1:** A partial list of the differentially expressed proteins involving energry metabolism and skeletal muscle in Luxi beef cattle (*MSTN*^*−/−*^ vs WT)

Protein accession	Protein description	Protein name	Fold-change ± SD (*MSTN*^*-/-*^vsWT)	*p*-value	Regulated
**Muscle related proteins**					
41386691	myosin-1	MYH1	1.53 ± 0.18	0.0001	Up
741980538	PREDICTED: troponin T, fast skeletal muscle isoform X32	TNNT3	5.62 ± 2.21	0.0300	Up
78369256	LIM domain-binding protein 3	LDB3	2.03 ± 0.31	0.0002	Up
261245063	myosin-2	MYH2	0.72 ± 0.05	0.0027	Down
741954711	PREDICTED: troponin I, slow skeletal muscle isoform X1	TNNI1	0.69 ± 0.13	0.0120	Down
77735655	troponin C, slow skeletal and cardiac muscles	TNNC1	0.71 ± 0.09	0.0071	Down
41386711	myosin-7	MYH7	0.77 ± 0.10	0.0181	Down
**Energy metabolism related proteins**					
28461197	glycogen phosphorylase, muscle form	PYGM	1.37 ± 0.23	0.0082	Up
114051459	fructose-1,6-bisphosphatase isozyme 2	FBP2	1.53 ± 0.20	0.0003	Up
156120479	fructose-bisphosphate aldolase A	ALDOA	1.34 ± 0.07	0.0016	Up
116004023	phosphoglucomutase-1	PGM1	1.48 ± 0.07	4.1E-05	Up
94966765	glucose-6-phosphate isomerase	GPI	1.39 ± 0.08	0.0010	Up
77736349	beta-enolase	ENO3	1.31 ± 0.10	0.0106	Up
77735551	phosphoglycerate kinase 1	PGK1	1.45 ± 0.10	0.0015	Up
77404273	glyceraldehyde-3-phosphate dehydrogenase	GAPDH	1.31 ± 0.06	0.0011	Up
61888856	triosephosphate isomerase	TPI1	1.31 ± 0.09	0.0051	Up
27806559	L-lactate dehydrogenase A chain	LDHA	1.63 ± 0.17	0.0003	Up
329664500	pyruvate kinase	PKM	1.31 ± 0.13	0.0057	Up
297493013	PREDICTED: phosphorylase b kinase regulatory subunit alpha, skeletal muscle isoform isoform X5	PHKA1	1.50 ± 0.22	0.0135	UP

Note: Corrected *p*-value ≤ 0.05, Fold change ≥ 1.30 or ≤ 0.77.

### Identification of differentially expressed phosphorylated peptides and proteins (DEPPs) between *MSTN*^*-/-*^ and WT

The results from the parallel global phosphoproteomics analysis in *MSTN*^*−/−*^ compared to WT cattle are summarized in Figure 1B. A total of 2,600 phosphopeptides containing 2,926 phosphosites from 702 phosphorylated proteins were identified, in which 149 differentially expressed phosphopeptides belonging to 76 DEPPs were confidently quantified with a fold change value of *MSTN*^*−/−*^/WT at 1.3 and a *p*-value ≤ 0.05 (see Supplementary Tables 2A, 2B). Among the 149 differentially expressed phosphopeptides, 81 up-regulated phosphopeptides belong to 41 phosphoproteins and 68 down-regulated phosphopeptides belong to 39 phosphoproteins (Figure 1B). Four phosphoproteins (e.g. myosin-1, sarcoplasmic reticulum calcium-binding protein, striated muscle kinase isoform X3 and myosin-binding protein C isoform X1 ) containing both increased and decreased abundance of unique phosphopeptides (see Supplementary Table 2B) were counted as both up-regulated and down-regulated proteins for subsequent GO analysis. The functions of 41 phosphoproteins were classified into the categories of glycometabolism related proteins (32%), muscle related proteins (37%), purine metabolism related proteins (7%). The functions of 39 phosphoproteins were classified to the categories of glycometabolism related proteins (5%), muscle related proteins (59%) (Supplementary Figures 1C, 1D). Therefore, the DEPPs were mainly involved in glycometabolism and muscle growth and development, which is in good agreement with the function of the DEPs.

Of the 2,926 phosphosites identified in this work (Supplementary Table 2A), 2,444 (83.5%) were phosphorylated at serine (pSer), 375 (12.8%) at threonine (pThr), and 107 (3.7%) at tyrosine (pTyr) residues. The results were similar to previous reports in rat: 88.1% for pSer, 11.4% for pThr and 1.5% for pTyr [43–45]. Of the 2,600 phosphopeptides, 2,303 (88.6%), 267 (10.3%) and 30 (1.1%) were found to be singly, doubly, and multiply phosphorylated peptides, respectively (Figure 1B).

**Figure 2:**
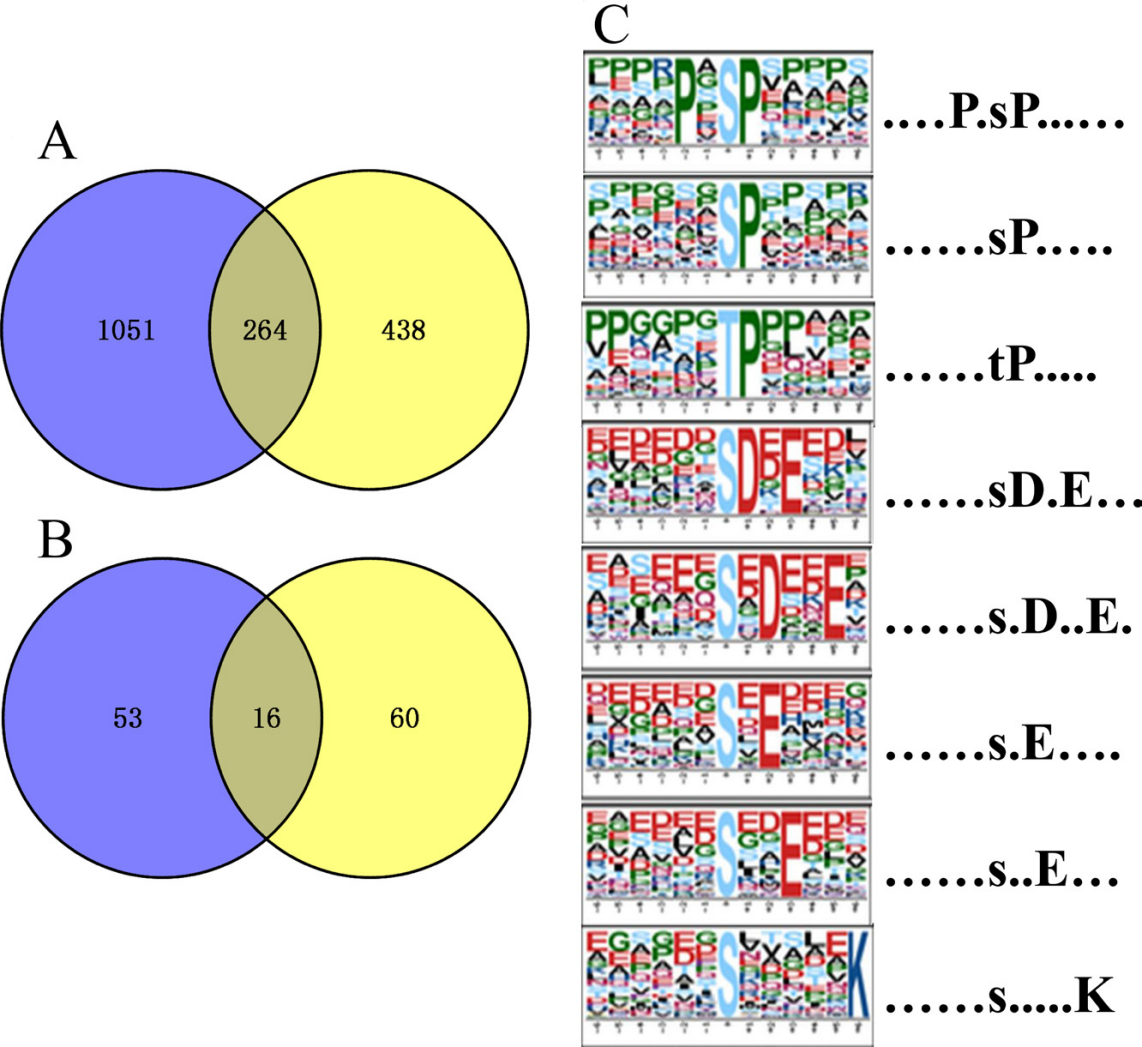
Venn diagrams of number of proteins/phosphoproteins identified in parallel proteomics and phosphoproteomics, and motif analysis of all the identified phosphosites (**A**) The number of quantified proteins and phosphoproteins; (**B**) the number of differentially expressed proteins and phosphoproteins; blue represent proteomics and yellow denote phosphoproteomics. (**C**) Significantly enriched phosphorylation motif of *MSTN*^*−/−*^ vs WT.

To compare the number of identified∕differentially expressed proteins and phosphorylated proteins, all the proteins found in proteomics (1,315) and phosphoproteomics (702) sets were shown in a Venn diagram. In both datasets we found 438 phosphorylated proteins, ∼62.4% of the total identified phosphoproteins were actually not identified in the global proteomics analysis (Figure 2A), indicating that majority of identified phosphoproteins are of relatively low abundance and identified mainly relying on the phosphopeptide enrichment by TiO2. Meanwhile, 60 out of a total 76 DEPPs (∼79%) were not found in the DEPs (Figure 2B), suggesting that a high percentage of phosphorylated proteins of low abundance are probably associated with regulation by *MSTN*. Motif-x analysis showed that seven phosphorylation motifs were enriched in the phosphoproteome of *MSTN*^*−/−*^ versus WT (Figure 2C). Acidic motifs including [sDxE], [sxE], [sxDxxE] and [sxxE] are recognized by Casein kinase II (CKII), which are involved in cell cycle and metabolic pathways [46, 47]. Motifs [PxsP], [sP] and [tP], the most common proline-directed motifs, are the substrates for mitogen-activated protein kinase (MAPK), ERK1, ERK2 Kinase [47, 48], and one basic motif [sxxxxxK] was not found in the referred phosphorylation database.

### Functional analysis of differentially expressed proteins and phosphorylated proteins

**Figure 3:**
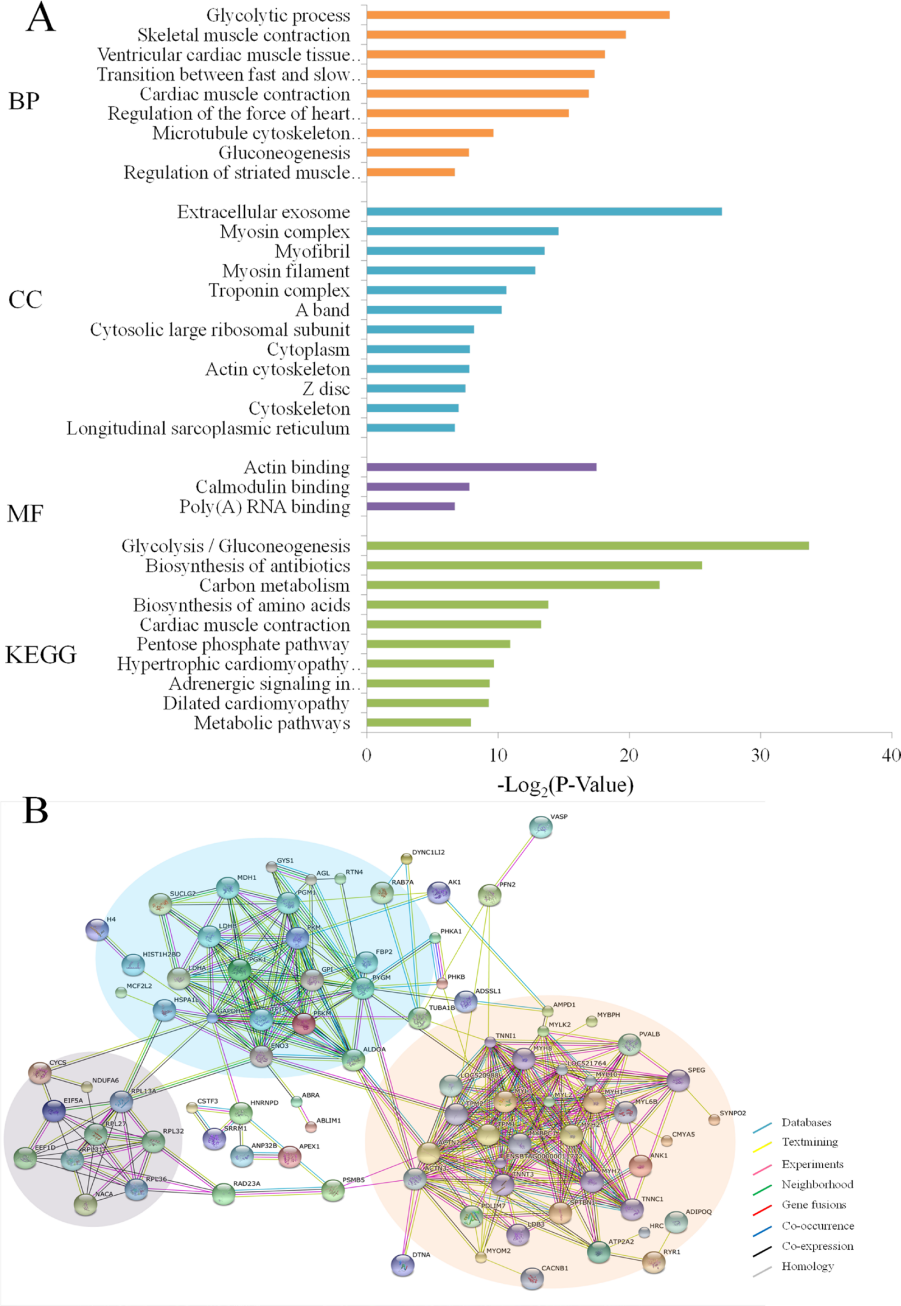
Functional classification of differentially expressed proteins and phosphoproteins in *MSTN*^−/−^ vs WT (**A**) GO and KEGG analyses. BP: Biological process, CC: Cellular component, MF: Molecular function, KEGG: Kyoto encyclopedia of genes and genomes. The values given in each of enriched terms or pathways are corrected *p* ≤ 0.01. (**B**) Protein-protein interaction analysis.

The results of GO analysis are shown in Figure 3A with *p* ≤ 0.01 as a significant threshold. Among the GO annotations and KEGG analysis of 129 differentially expressed proteins and phosphorylated proteins, the glycolytic process, extracellular exosome and actin binding were significantly enriched as the top categories of biological process (BP), cellular component (CC) and molecular function (MF), respectively. Meanwhile, Glycolysis/glycogenolysis pathways were also remarkably enriched against the current KEGG database. These results of GO analysis strongly suggest that regulation by the *MSTN* gene affected mainly glycolysis and glycogen metabolism in muscle tissue and muscle development. Further analysis of putative protein interaction networks for those changed proteins using STRING database revealed three major protein-protein interaction networks among the set of 129 differentially expressed proteins (Figure 3B). The largest network, highlighted by an orange background in Figure 3B, includes more than 30 proteins (e.g. myosin light chain kinase 2 and parvalbumin alpha) that are known to be associated with muscle development. The second network highlighted with a blue background contains ∼20 proteins including phosphorylase b kinase regulatory subunit alpha, and is mainly involved in metabolism and the third network includes ∼10 proteins (e.g. 60S ribosomal protein L31) that function in muscle translation. These findings are essentially similar to the results found from GO analysis.

To test the possibility that the DEPPs identified in the phosphoproteome may function differently from the DEPs in the global proteome or if any particular functional classes of proteins are preferentially becoming phosphorylated, both GO and STRING analyses were also conducted on 69 DEPs and 76 DEPPs separately. Consistent results were obtained for both DEPs and DEPPs (Supplementary Figure 2), despite the fact that only a small portion (20–30%) of the proteins are overlapped between the two sets.

### Verification of the *MSTN* regulated proteins using MRM and Western blot analysis

**Figure 4:**
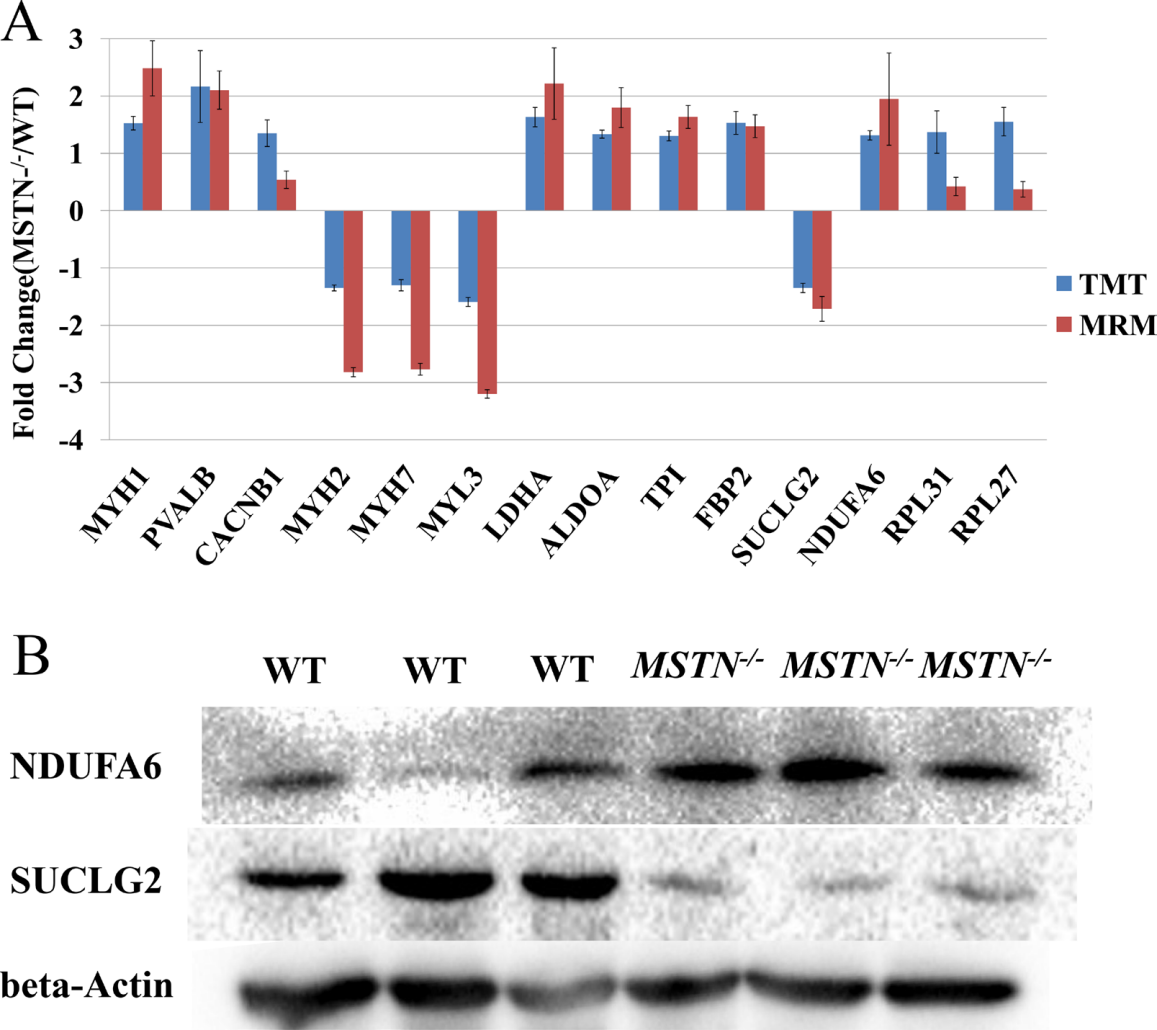
Comparison and verification of the quantitative results for differentially expressed proteins from TMT-6 plex proteomics quantitation, MRM and Western blot analyses (**A**) The results of fourteen DEPs from the TMT 6-plex proteomics quantitation and MRM analyses. (**B**) Western blot result of NDUFA6 and SUCLG2 proteins. These blots were cropped and original images were shown in Supplementary Figure 3.

MRM using a triple quadrupole mass spectrometer is considered to be one of the most accurate, sensitive and reproducible methods for quantitation of targeted proteins or peptides [49]. To verify the TMT-based quantitative proteomics results, we used both MRM and classical Western blot analyses to cross-validate the expression of proteins whose abundance changed in response to *MSTN*^*−/−*^. Fourteen DEPs of interest (Supplementary Tables 3A, 3B) were selected for MRM analyses, covering those mainly involved in muscle development and energy metabolism. Through statistical analyses, we found that the ratio of *MSTN*^*−/−*^ to WT in the MRM data showed a similar trend with the TMT analyses, and direct comparisons of relative quantitation between *MSTN*^*−/−*^ and WT triplicate samples for the 14 selected proteins are shown in Figure 4A. To further confirm the results, given the availability of some antibodies, two DEPs (NDUFA6 and SUCLG2) and beta-actin (for internal control) were tested for Western blot analyses. As shown in the Figure 4B, the fold changes (*MSTN*^*−/−*^/WT) determined by WB, MRM and TMT are ∼5.26, 1.95 and 1.31 respectively for NDUFA6, and 0.53, 0.53 and 0.74 respectively for SUCLG2, demonstrating the quantitative proteomics data is reasonably accurate.

### *MSTN*^*-/-*^ induces abundance and/or phosphorylation changes of proteins in the glycogenolysis and glycolysis pathways

**Figure 5:**
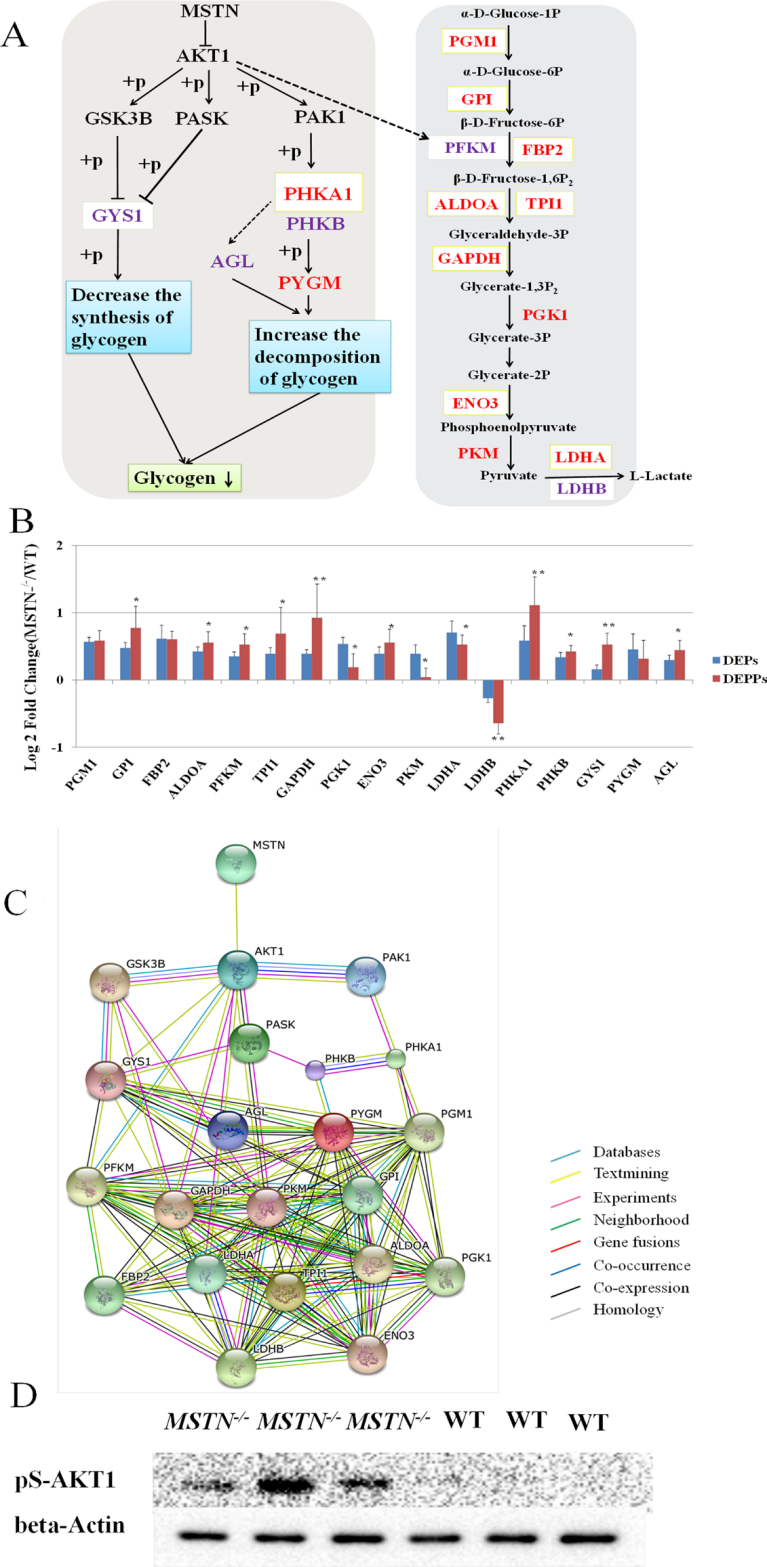
Functional interaction network and a diagrammatic mode illustrate the regulatory mechanism of *MSTN* gene on the glycogenolysis and glycolysis pathways (**A**) “→” The activation of the process, “ -| ” the inhibition of the process, “– –” the presence of intermediate steps either unknown or omitted. The DEPs were marked in red font, DEPPs were marked in purple font, and the shared proteins were labeled with a light yellow box. Glycogenolysis was highlighted by an orange background, and glycolysis was highlighted with a blue background. (**B**) Seventeen DEPs/DEPPs in both proteomics and phosphoproteomics data involved in glycolysis and glycogenolysis pathways respectively were identified and validated. (**C**) MSTN, Akt1, and 17 DEPs/DEPPs were submitted to conduct blast searching against the existing databases in STRING 10 software. (**D**) Western Blot result of pAkt1 (Ser-473), demonstrating pAkt1 (Ser-473) is up-regulated in *MSTN*^*−/−*^than WT. These blots were cropped and original and original images were shown in Supplementary Figure 4.

For identified DEPs and DEPPs, we observed a group of them (12 DEPs and 14 DEPPs) associated with glycolysis and glycogenolysis pathways in *MSTN* disruption (Figure 5A, Tables 1 and 2). Strikingly, all 12 DEPs and 13 out of 14 DEPPs (Figure 5A) showed an increased abundance in the *MSTN*^*−/−*^ transgenic cattle vs WT. Two DEPs including glycogen phosphorylase (PYGM, FC = 1.37), phosphorylase b kinase regulatory subunit alpha (PHKA1, FC = 1.50) are involved in glycogen degradation, while the remaining 10 DEPs are involved in almost all 10 steps of the glycolysis pathway. Four DEPPs including phosphorylase b kinase regulatory subunit beta (PHKB, FC = 1.34), glycogen debranching enzyme (AGL, FC = 1.36), PHKA1, FC = 2.16 and glycogen synthase (GYS1, FC = 1.44) are found to directly participate in glycogen metabolism by increasing glycogen decomposition and decreasing glycogen synthesis (Figure 5A). Similarly, the remaining 10 DEPPs including ATP-dependent 6-phosphofructokinase (PFKM, FC = 1.44), L-lactate dehydrogenase B chain (LDHB, FC = 0.64) along with the other 8 enzymes (FC ≥ 1.30) found in the same DEPs, are also involved in the glycolysis pathway. Further comparison of up-regulated PHKA1 and 8 glycolysis enzymes found in *MSTN*^*−/−*^ with increased abundance in both proteomics and phosphoproteomics data is shown in Figure 5B, we found that the 8 glycolysis enzymes revealed a very similar degree of increased expression, whereas PHKA1 showed a significantly higher increase of abundance in the phosphorylated form (FC = 2.16) than the non-phosphorylated form (FC = 1.44), suggesting that regulation of PHKA1 for glycogen metabolism by phosphorylation appears important in *MSTN*^*−/−*^ cattle. In addition, we found the PFKM, which catalyzes a rate-limited step of glycolysis, showed an increased abundance of its phosphorylated form (FC = 1.44), but no significant change was observed in native form between *MSTN*^*−/−*^ and WT beef cattle, also indicating the importance of phosphorylation regulation on glycolysis. Nevertheless, these 12 DEPs and 14 DEPPs suggest that both glycogenolysis metabolism and glycolysis were considerably impacted upon *MSTN* disruption. A STRING protein network consisting of all glycogenolysis/glycolysis associated proteins of interest in this study is shown in Figure 5C. Western blotting for pAkt expression was also conducted for verification of proposed PI3K/Akt activation as a possible upstream regulation of PHKA1 and GYS1. The result showed that remarkably higher expression of pAkt was detected in *MSTN*^*−/−*^ than WT gluteus (Figure 5D).

**Table 2:** A partial list of the differentially expressed phosphoproteins involving engery metabolism and skeletal muscle in Luxi beef cattle (*MSTN*^*-/-*^ vs WT)

Protein accession	Protein description	Protein name	Site	Phosphopeptide sequence	Fold-change±SD (*MSTN*^*-/-*^vsWT)	*p*-value	Regulated
*529014933*	*PREDICTED: phosphorylase b kinase regulatory subunit alpha, skeletal muscle isoform isoform X6*	*PHKA1*	*s*	*rLsISTESQVk*	*2.16* ± 0.42	*0.010*	*Up*
358416561	PREDICTED: LOW QUALITY PROTEIN: phosphorylase b kinase regulatory subunit beta	PHKB	t	rQSStSnAPEQEQkPDVTMTEWR	1.34 ± 0.09	0.003	Up
*155372313*	*glycogen [starch] synthase, muscle*	*GYS1*	*s*	*yPRPAsVPPsPSLSR*	*1.44* ± 0.17	*0.007*	*Up*
115497288	ATP-dependent 6-phosphofructokinase, muscle type	PFKM	s	kNVLGHmQQGGsPTPFDR	1.44 ± 0.16	0.012	Up
115497288	ATP-dependent 6-phosphofructokinase, muscle type	PFKM	s	gRsFmnNWEVYk	1.49 ± 0.09	0.001	
300794727	glycogen debranching enzyme	AGL	t	yTWtDVGQLVQk	1.36 ± 0.14	0.011	Up
114051459	fructose-1,6-bisphosphatase isozyme 2	FBP2	y	iYSLNEGyAk	1.52 ± 0.12	0.002	Up
156120479	fructose-bisphosphate aldolase A	ALDOA	s	gILAADEsTGSIAk	1.47 ± 0.16	0.007	Up
116004023	phosphoglucomutase-1	PGM1	y	lYIDSyEkDLAk	1.50 ± 0.15	0.002	Up
94966765	glucose-6-phosphate isomerase	GPI	t	iEPELDGSSPVtSHDSSTNGLINFIk	1.71 ± 0.32	0.029	Up
77736349	beta-enolase	ENO3	s	eILDsRGNPTVEVDLHTAk	1.47 ± 0.20	0.010	Up
27806559	L-lactate dehydrogenase A chain	LDHA	y	qVVDSAyEVIk	1.44 ± 0.14	0.003	Up
77404273	glyceraldehyde-3-phosphate dehydrogenase	GAPDH	t	vIHDHFGIVEGLMTTVHAITAtQk	1.90 ± 0.50	0.029	Up
61888856	triosephosphate isomerase	TPI1	s	eLAsQPDVDGFLVGGASLkPEFVDIINAk	1.61 ± 0.39	0.038	Up
27806561	L-lactate dehydrogenase B chain	LDHB	s	iVADkDYsVTANSk	0.64 ± 0.16	0.029	Down
77736203	malate dehydrogenase, cytoplasmic	MDH1	s	nVIIWGNHSsTQYPDVNHAk	0.67 ± 0.09	0.001	Down

Note: Corrected *p*-value ≤ 0.05, Fold change ≥1.3 or ≤ 0.77.

### Sequence alignment analysis, ELISA verification and muscle glycogen content measurement

**Figure 6:**
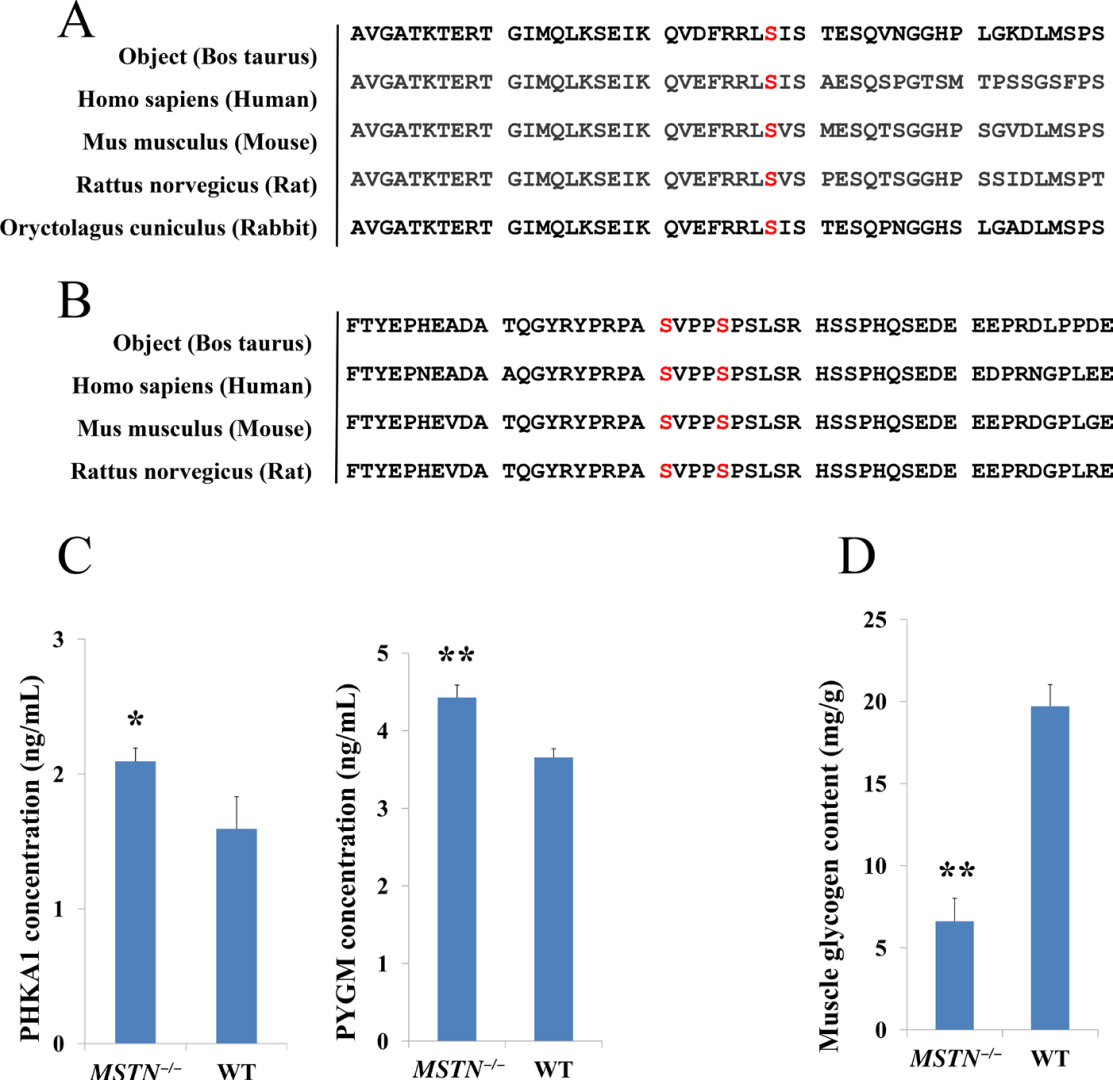
Glycogenolysis genes validation and muscle glycogen content measure (**A**, **B**) Amino acid sequence alignment of PHKA1 and GYS1 between Bos Taurus and other species indicates that S1018 residue of PHKA1 is conserved and, S641 and S645 of GYS1 are conserved among other important animal species, suggesting the phosphorylation of S1018 residue in PHKA1 and S641 and S645 residue in GYS1 protein may play an important regulatory role in glycogen catabolism under *MSTN* disruption. (**C**) ELISA verification of PHKA1 and PYGM in both *MSTN*^*−/−*^ and WT beef cattle, the results shown that PHKA1 and PYGM were significantly up-regulated, which are consistent with phosphoproteomics data. (**D**) Muscle glycogen contents in *MSTN*^*−/−*^ and WT beef cattle.

In comparative phosphoproteomics analysis, one PHKA1 phosphopeptide “1016-RLsISTESQVk-1026”, containing phosphorylation at serine-1018 residue (Ser-1018) and one GYS1 doubly phosphorylated peptide “636-YPRPAsVPPsPSLSR-650” at Ser-641 and Ser-645 were confidently identified with a 2.16-fold and 1.44-fold increase in abundance, respectively in *MSTN*^*−/−*^ than WT (Table 2). To examine the importance of those phosphorylated sites in the function of PHKA1 and GYS1, sequence alignment of PHKA1 between *Bos taurus* and four most common species (e.g. Human, Mouse, Rat and Rabbit) were performed. The result shows that Ser-1018 residue is indeed conserved in these 5 species (Figure 6A). Interestingly, the Ser-1018 of PHKA1 in rabbit was reported as a phosphorylation site [50–52], consistent with our result for Ser-1018 phosphorylation of PHKA1 in *Bos taurus.* Similarly, sequence alignment of GYS1 between *Bos taurus* and three common species, also demonstrated that both Ser-641 and Ser-645 are conserved in these species (Figure 6B). Interestingly, it was reported that phosphorylation at Ser-641 of GYS1 by DYRK2 or PASK resulted in GYS1 inactivation, while dephosphorylation at Ser-641 and Ser-645 by PP1 activated the GYS1 enzyme [53]. Thus, the increased abundance in phosphorylation at Ser-641 and Ser-645 of GYS1 found in our study is expected to inactivate the GYS1 enzyme and therefore to inhibit glycogen synthesis.

The abundance changes of PHKA1 protein and its downstream PYGM were validated by ELISA analysis. As shown in Figure 6C, both PHKA1 and PYGM proteins were significantly up-regulated in *MSTN*^*−/−*^/WT. This result further confirmed the good agreement with proteomics and phosphoproteomics data sets for both PHKA1 and PYGM. To further assess if PHKA1 and GYS1 phosphorylation has any effect on the muscle glycogen content after *MSTN* disruption, we measured the glycogen content of *MSTN*^*−/−*^ and WT cattle gluteus. The result showed that the glycogen content decreased remarkably in *MSTN*^*−/−*^ set (6.6 mg/g muscle tissue) compared to WT set (19.7 mg/g muscle tissue) (Figure 6D).

## DISCUSSION

In this study, we presented complementary results on the parallel global proteomics and phosphoproteomics changes in gluteus muscle between *MSTN*^−/−^ and WT cattle to investigate the global regulatory mechanism of *MSTN* related to cellular metabolism. Three factors sparked our interest in conducting this study. One is that previous genomic and transcriptomic studies showed that all *MSTN* regulated signaling cascades (e.g. Wnt4/β-catenin [16], IGF-I pathway [17], MAPK pathway [18] and PI3K/Akt pathway [17, 19]) leading to skeletal muscle atrophy, are involved in reversible phosphorylation regulation by kinases and phosphatases. The second factor is that to date as far as we are aware, no global phosphoproteomics changes in muscle tissue of *MSTN*^*−/−*^ animals have been investigated although a few studies of the global proteomics level in *MSTN*^−/−^ mice being recently reported [28, 34]. Lastly, many of the existing studies have focused on small animal models, in this study, we take an advantage of the availability of the transgenic *MSTN*^*−/−*^ cattle to our group allowing us to use as a heavy livestock animal model to study the function of *MSTN* gene in animals with high economics impact, which can provide a theoretical basis for the biological safety and genetic breeding of *MSTN* gene editing cattle. We conducted careful cross-method verification analyses to validate the proteomics data using MRM and traditional western blotting. The MRM data demonstrated that the 14 selected DEPs were consistent with the fold changes from global proteomics (Figure 4A and Supplementary Table 3B). An additional Western blotting analysis on both NDUFA6 and SUCLG2 proteins selected from the MRM-validated group further confirmed the significant up-regulation of NDUFA6 and down-regulation of SUCLG2 in *MSTN*^−/−^ (Figure 4B). Based on these results, we are confident that the global quantitative data presented in this work are reproducible and reliable. It should be noted that our TMT-based global proteomics results (1,315/69) for *MSTN* transgenic and WT cattle samples is in good agreement with the recent report (1137/136) for SILAC-based proteomics on *MSTN*^*−/−*^ knockout mice [34]. The relative low number of DEPs found in our study may reflect the combined factors for different *MSTN*^*−/−*^ genotype status with different species, and possible ratio compression caused by the MS2-based TMT quantitation technique [41].

In the set of DEPs and DEPPs related to skeletal muscle structure proteins, our data show that relatively fewer proteins associated with fast-twitch fibres such as myosin-1, Troponin T3 (TNNT3) and LIM domain-binding protein 3 (LDB3), revealed a dramatically increased abundance whereas more proteins associated with slow-twitch fibres such as myosin-2, Troponin I/C and myosin-7, had a decreased abundance in *MSTN*^-/-^ (Table 1, Supplementary Table 1B). All these classes of DEPs play an important role in binding calcium for muscle contraction and many physiological processes in the cell. For example, TNNT3 specific for fast-twitch muscle was found to have the largest fold-change (FC = 5.6) in *MSTN*^-/-^ and LDB3 (FC = 2.2) responsible for structural integrity of sarcomeres during contraction, and it has been shown to be involved in protein kinase A signaling [54]. Several troponin isoforms specific for slow-twitch muscle were significantly decreased in *MSTN*^-/-^. These results are in good agreement with the recent proteomics report for *MSTN*^-/-^ mice [34] and support that *MSTN* disruption leads to a slow-to-fast fibre switch. In the sets of DEPs and DEPPs related to energy metabolism, the majority of up-regulated proteins were key enzymes in glycolysis and glycogenolysis pathways (Figure 5, Tables 1 and 2), suggesting that glycolysis and glycogenolysis were considerably enhanced, which indicated that the capacity for muscle energy supply might be increased. Conversely a few mitochondria proteins including succinyl-CoA ligase, a key catalyst involved in the citric acid cycle, were significantly down-regulated, suggesting a decrease of oxidative energy metabolism may be associated with *MSTN* gene disruption. These findings support an apparent anaerobic glycolytic shift for the switch from slow-twitch to fast-twitch muscle that would considerably compromise strength production in association with anticipated reduction of oxidative energy metabolism within skeletal muscle in *MSTN*^*−/−*^ cattle and are consistent with the previous reports in the double-muscle animals [16, 28, 41].

Five proteins involved in glycogen metabolism including phosphorylase b kinase regulatory subunit alpha (PHKA1) and subunit beta (PHKB), glycogen phosphorylase (PYGM) and glycogen debranching enzyme (AGL) for glycogenolysis, and glycogen synthase (GYS1), responsible for glycogen metabolism were upregulated. PHKA1 is a key regulatory kinase catalyzing the phosphorylation of serine in targeted proteins such as troponin I, while the beta subunit in PHKB acts as a regulatory unit and modulates the activity of the holoenzyme in response to upstream phosphorylation. PYGM catalyzes the rate-limiting step in glycogenolysis by releasing glucose-1-phosphate (G1P) from the terminal alpha-1,4-glycosidic bond. AGL then transfers three of the remaining four glucose units to the end of another glycogen branch to be further metabolized in the glycolytic pathway. Thus it is conceivable that increased abundance of those 5 proteins in the *MSTN*^*−/−*^ cattle compared to WT will facilitate glycogen decomposition and suppress glycogen synthesis (Figure 5A). As a result, glycogen as a storage form of glucose and a major energy substrate can supply G1P for glycolysis in muscle cells during muscle contraction and growth.

We take advantages of the parallel analysis design, which allows one for dissecting changes in phosphorylation state from changes in protein abundance, and therefore for enhanced confidence in identifying potential sites of phosphoregulation, when the two omic data sets are integrated. The comparison results of the abundance change alone on all 17 DEPs/DEPPs (Figure 5B) indicate that 14 out of 17 enzymes exhibit significant difference between DEPs and DEPPs, suggesting phosphorylation on most metabolic enzymes appear selective (e.g. up-regulated for PFKM and down-regulated for PKM) by either upstream kinases or phosphatases. Two phosphorylated peptides of PFKM: 372—LRGRsFMNNWEV—383 (FC = 1.49) and 663—QQGGsPTPFDRN—674 (FC = 1.44) as shown in Table 2, were increased in abundance in *MSTN*^*−/−*^ and also conserved in human, rat and mouse [44, 45, 55]. Those phosphorylated sites identified particularly on the limiting step enzymes could be used for further investigation by *in vitro* mutation and subsequent function analysis. Interestingly, an increase in pAkt signaling in *MSTN*^*−/−*^ cattle (Figure 5D) also supports with an increase in glycolysis metabolism as Akt was reported to indirectly activate phosphorylation of phosphofructokinase-2 (PFK2) which activates phosphofructokinase-1 (PFK1) [56]. It is surprising that there is no abundance difference between phosphorylated PYGM versus its unmodified form (Figure 5B) as PYGM is an important rate-limiting step in glycogenolysis. However, PYGM is a well-known model protein regulated by reversible phosphorylation and allosteric effects, our data suggest that allosteric effects in PYGM may play more profound roles once reversible phosphorylation occurs. One of the striking findings is that both PHKA1 and GYS1 show remarkably higher abundance in their phosphorylated form (2.16 and 1.44 respectively) than their unmodified form (1.50 and 1.11 respectively) in *MSTN*^*−/−*^ transgenic versus WT cattle, suggesting that the up-regulation of PHKA1 and GYS1 by phosphorylation on glycogen metabolism seems important in *MSTN*^*−/−*^ cattle. Thus both PHKA1 and GYS1 as key phosphorylated protein candidates were further investigated for the effect of their phosphorylation sites and upstream regulation on glycogen metabolism in *MSTN*^*−/−*^ cattle.

As the major storage form of energy, glycogen is readily mobilized during muscle growth and development. Systemic glycogen synthesis and breakdown are primarily regulated by insulin signaling pathway [57]. In the *MSTN*^*−/−*^ animals however, insulin signaling (determined by Akt phosphorylation) was significantly elevated [58]. Our Western blotting with anti-phospho-Akt (Ser-473) confirmed the dramatically increased expression of pAkt in *MSTN*^*−/−*^ cattle (Figure 5D). When Akt phosphorylation is activated, it regulates the downstream kinases (GSK3 and PASK) to affect GYS1 activity on glycogen metabolism (Figure 5A). Phosphorylation at tyrosine-216 in GSK3β or tyrosine-279 in GSK3α enhances GSK3 activity, whereas phosphorylation of serine-9 in GSK3β or serine-21 in GSK3α significantly decreases GSK3 activity [59]. It has been reported that when GSK3 is maximally inhibited, it results in an increased glycogen synthesis [60] by regulating GYS1 phosphorylation. The phosphorylation occupancy rate of the different sites in GYS1 played an important role in regulation of its activity. For example, *in vitro* phosphorylation at Ser-8 of purified GYS1 from rabbit skeletal muscle by AMPK or phosphorylation at Ser-641 by Per-Arnt-Sim Kinase (PASK) inactivates GYS1 activity, while dephosphorylation at Ser-641 and Ser-645 by protein phosphatase (PP1) activates the enzyme [53]. In this paper, we found the phosphorylated peptide (636—YPRPAsVPPsPSLSR—650) of GYS1 (Ser-641 and Ser-645), was up-regulated (FC = 1.44) but no change of GYS1 abundance in *MSTN*^*−/−*^ versus WT cattle. Based on conserved sequences for both the 641 and 645 sites across 5 species including rabbit [61–64] (Figure 6B), and previous *in vitro* results, we suspect that the increased abundance of phosphorylation on S641 and S645 of GYS1 in *MSTN*^*−/−*^ cattle will most likely result in lower GSY1 activity and therefore suppress glycogen synthesis. Notably, our phosphoproteomics data showed a confident identity of the Tyr-216/279 phosphorylated peptide (GEPNVSyIcSR) from GSK3, and its Ser-9/21 phosphorylated peptide (TSsFAEPGGGGGGGGGGPGGSASGPGGSGGGK) (Supplementary Table 2A) despite the fact that GSK3 was not identified in our global proteomics. This finding suggests GSK3β/α, as an upstream kinase of GYS1 is of relatively low abundance but likely play a dynamic regulatory role in GYS1 activity (Figure 5A).

Phosphorylase b kinase (PHK) regulates glycogen metabolism via activating glycogen phosphorylase activity, which promotes glycogen breakdown [65]. The PHK holoenzyme contains three distinct regulatory subunits, (α/β/δ), and one catalytic subunit, and two regulatory subunits (α/β) were skeletal muscle isoform [65]. PHKA1 activates the downstream PYGM by phosphorylation and accelerates glycogen breakdown. We observed an increased abundance of PHKA1 phosphorylation and PYGM in which phosphorylation by PHKA1 is likely to be involved in energy metabolism and muscle contractile fibre process, but also regulates allosteric interaction that could play a key role as discussed above. The phosphorylation site (Ser-1018) of PHKA1 (1016—RLsISTESQVK—1026) is a major phosphorylation regulatory site in PHKA1 [50] and is highly conserved across species (Figure 6A). PHKA1 phosphorylation was catalyzed by the upstream kinase PAK (p21-activated kinases). The PAK along with GSK and PASK was activated by phosphorylation by Akt kinase [66] in the PI3K/Akt signaling pathway (Figure 5A). Nevertheless, our data suggest that on the one hand, increased phosphorylation (Ser-641 and Ser-645) of GYS1 by upstream kinases (GSK and PASK) appears to inhibit activity of glycogen synthase; on the other hand, an increased PHKA1 phosphorylation (Ser-1018) (by upstream kinase PAK1) and other glycogenolysis regulation proteins (PHKB and AGL) likely promote faster glycogen breakdown in *MSTN*^*−/−*^ over WT. Therefore we expect to have a considerable reduction of muscle glycogen in *MSTN*^*−/−*^ compared to WT skeletal muscles and our biochemical measurement results indeed demonstrate this. More than a 3-fold glycogen reduction was found in *MSTN*^*−/−*^ versus WT as shown in Figure 6D.

In addition, there is a high correlation between the types of muscle fibre and the urgency of required energy metabolism. *MSTN*^*−/−*^ beef cattle possess a higher number of fast fibres than WT beef cattle [5, 67], and our proteomics and phosphoproteomics data show a slow-to-fast fibreswitch observed in *MSTN*^*−/−*^. This switch provides an additional piece of evidence that fast muscle fibre readily acquired energy via glycolytic metabolism, so that the body must accelerate glycogenolysis metabolism and have sufficient G1P ready for subsequent glycolysis. In summary, our results reveal that enhanced glycogenolysis and glycolysis metabolism with decreased oxidative characteristics in the skeletal muscle support the conclusion that *MSTN* plays a critical role in muscle development and energy metabolism.

Motif analysis of phosphopeptides obtained through phosphoproteomics data sets is often used to predict the protein kinases responsible for the phosphorylation. In good agreement with previous reports [46–48, 68], our phosphoproteomics data also show that some important energy metabolism related regulatory proteins or kinases (e.g. GYS1, PHKA1, PHKG1 and PPP1R2) and MAPK signaling pathway related proteins or kinases (e.g. MAP2K6, MAPT, FLNA and FLNC) were respectively phosphorylated at acidic casein kinase II motifs and Pro-directed motifs, suggesting these proteins are potential substrates of the corresponding kinases. Given the identified motifs and combining with known Serine/Threonine kinases in consensus phosphorylation site specificity, we are able to predict the upstream kinases for some of the important targeted proteins involved in the glycogenolysis metabolism and glycolysis pathways that are regulated by *MSTN*^*−/−*^ signaling cascades. For instance, the phosphorylated peptide identified in cattle GYS1 at both S641 and S645 sites (636—YPRPAsVPPsPSLSR—650) contains a specific motif [sxxxsP] for Glycogen Synthase Kinase 3 (GSK3) that is regulated through phosphorylation by upstream protein kinase B (PKB/Akt) [69] plus the increased abundance of pAkt was confirmed in *MSTN*^*−/−*^ by Western blotting in (Figure 5D). Thus, possible GYS1 inactivation by GSK3 and/or others (such as PASK) allows us to connect glycogenesis to the upstream PI3K/Akt signaling pathway that is one of the signaling cascades mediated by myostatin binding to its receptor ActRIIB [35]. A similar analysis was used for the phosphopepitde in PHKA1 with S1018 phosphosite (1015—R.RLsISTESQVK—1026) having a specific motif [RRxsxx] for PAK, that can be regulated by many upstream activators including Akt [66], ERK, CKII regulated in the PI3K/Akt signaling pathway.

Our study using TMT-based parallel comparative proteomics and phosphoproteomics analyses on skeletal muscle from *MSTN*^*−/−*^ transgenic and WT cattle, represents the first global phosphoproteomics investigation for *MSTN* regulatory mechanism. The integrated proteomics and phosphoproteomics data reveal that *MSTN* induces abundance changes of targeted proteins and phopshorylation of key enzymes in glycogen metabolism and glycolysis pathway. This study reveals that both the abundance increase of most enzymes and phosphorylation on their regulatory sites seem associated with the enhanced glycogenolysis and glycolysis pathways in *MSTN*^*−/−*^. A proposed myostatin signaling through PI3K/Akt pathway for increased glycogenolysis metabolism in skeletal muscle was supported by the data that the increased abundance of pAkt in *MSTN*^*−/−*^, and increased phosphorylation of regulatory sites at Ser1018 of PHKA1, and Ser641/Ser645 of GYS1, mediated respectively, by PAK and GSK3 under PI3K/Akt signaling pathway. By combination of the results reported in this work and previous studies we conclude that *MSTN* plays a critical role in muscle development and energy metabolism with elevated glycogenolysis and glycolysis pathways, and decreased oxidative characteristics.

## MATERIALS AND METHODS

### Experimental design and sample collection

The specific objective of this work was using our available *MSTN*^*−/−*^ cattle system to investigate the regulatory mechanism of MSTN related to metabolism in muscle development. TMT6-plex based parallel quantitative proteomics and phosphoproteomics analyses were designed and conducted in this study. A schematic diagram for experimental design and workflow is shown in Figure 1A.

This study was approved by Animal Welfare Committee of Inner Mongolia University of China. All experiments were carried out in strict accordance with the recommendations in the Guide for the Care and Use of Animals of Inner Mongolia University and approved by the Animal Welfare Committee of Inner Mongolia University.

*MSTN* gene editing Luxi beef cattle (treatment group; named as *MSTN*^-/-^) and wild type Luxi beef cattle (control group; named as WT) with a uniform growth stage (at age of 8–9 months) were obtained from animal experimental base, Inner Mongolia University of China. Three individual cattle in each group were randomly selected. The transgenic cattle *MSTN*^-/-^ was constructed by our collbrators in China Agricultural University and Inner Mongolia University using zinc finger nucleases technology [70].

Sampling technology of living specimen was used to collect tissue of cattle, which avoided to slaughter animals and reflected the living state of animals. Muscle tissues were collected from gluteus of each individual cattle for both groups. All 6 collected individual samples were immediately frozen in liquid nitrogen, and stored at -80°C until subsequent analysis.

### Protein extraction and digestion

Gluteus muscle tissues were crushed by conventional grinding method, and homogenized in RIPA lysis buffer (1% NP-40, 0.5% sodium deoxycholate, 1% SDS solution, 5 ml 1×PBS, 42 ml milliQ water) with PMSF and phosphatase inhibitors (RIPA: PMSF: phosphatase inhibitors was 100:1:1). The lysates were centrifuged 10 min at 12,000 g, 4°C, and the supernatants were transferred into a new tube. Quantitative analysis was performed using the BCA method. The proteins were lyophilized and stored at –80°C for further analysis.

The lyophilized proteins were resuspended and denatured for 5 min sonication and 0.5 hour of votex in 7 M urea, 2 M thiourea with a final concentration of 100 mM phosphate buffer (pH 7.8) containing 0.5 tablets PhosSTOP (Phosphatase Inhibitor Cocktail Tablets from Roche) for 5 mL buffer. The protein concentration for each of the 6 samples was quantified by a gel-based analysis as described previously [71]. The samples were reduced with 9.5 mM Tris-(2-carboxyethyl) -phosphine (TCEP), alkylated 17 mM iodoacetamide and quenched by additional of 20 mM Dithiothreitol (DTT). The alkylated proteins (200 µg) for each sample were precipitated in 6 volumes of ice-cold acetone at -20°C overnight, and reconstituted by adding 90 µL of 100 mM triethylammoniumbicarbonate. Each sample was digested with 10 μg trypsin for 18 h at 37°C.

### TMT6-plex labeling and high pH reverse phase LC (hpRPLC) Fractionation

The 6-plex tandem mass tag (TMT) labeling was carried out according to Thermo Scientific’s TMT Mass Tagging Kits protocol (http://www.piercenet.com/instructions/2162073.pdf). Each of the TMT 6-plex label reagents was reconstituted in 45 µL of acetonitrile (ACN), and the digested peptides from each sample were incubated with a specific tag (tags 126, 127, 128 used for 3 *MSTN*^*−/−*^ samples and tags 129, 130, 131 for 3 WT samples, respectively) for 1 hour in room temperature (Figure 1A). The efficiency of TMT labeling was assessed on nanoLC-Orbitrap Elite (Thermo-Fisher Scientific, San Jose, CA) by mixing 1 µL aliquots from each of the samples and desalting with SCX ziptip (Millipore, Billerica, MA). After labeling check, TMT tagged peptides from each sample were pooled, evaporated to dryness and cleaned up on a Waters Oasis MCX cartridge (1 mL × 30mg Oasis MCX, Waters, Milford, MA).

The hpRP chromatography was carried out as described previously [72]. Forty-eight fractions were obtained at 1 min intervals, and pooled into 10 fractions based on UV absorbance at 214 nm and with a multiple fraction concatenation strategy [73]. About 10% of the labeled tryptic peptides from each of the 10 fractions were used for global proteomics analysis by nanoLC-MS/MS analysis while the remaining 90% of samples in the 10 fractions were further pooled into 5 fractions and subjected to subsequent TiO_2_ enrichment for quantitative phosphoproteomics analysis.

### Enrichment of phosphopeptides by TiO2 beads

TiO2 enrichment was conducted using a TiO_2_ Mag Sepharose kit (from GE Healthcare). The TMT 6-plex tagged tryptic peptides were reconstituted in 400 µL of binding buffer (1 M glycolic acid in 80% acetonitrile, 5% TFA). The TiO2 slurry (75 µL) was used and incubated with the sample for 30 min at 1,800 rpm vortex. After washing the beads with washing buffer (80% acetonitrile, 1% TFA), the phosphopeptides were eluted with 100 µL of elution buffer (5% ammonium hydroxide) twice. The eluted fraction was dried and reconstituted in 25 µL of 0.5% formic acid (FA) for subsequent nano scale LC-MS/MS analyses.

### Nano-scale reverse phase chromatography and tandem MS (NanoLC-MS/MS) analysis

NanoLC-MS/MS analysis was carried out using an Orbitrap Fusion mass spectrometer (Thermo-Fisher Scientific, San Jose, CA) with an UltiMate3000RSLCnano system (Thermo-Dionex, Sunyvale, CA). The Orbitrap Fusion was interfaced with nanospray Flex Ion Source using high energy collision dissociation (HCD) similar to previous reports [71, 72]. Each reconstituted fraction was injected onto a PepMap C18 RP nano trapp column (3 µm, 100 µm×20 mm, Dionex) with nanoViper Fittings at 20 µL/min flow rate, and separated on a PepMap C18 RP nano column (3 µm, 75 µm × 25 mm, Dionex) with the detailed gradient as previously report [74]. The eluted peptides were analyzed on the Orbitrap Fusion, which was operated in position ion mode with a spray voltage set at 1.6 kV and the source temperature at 275°C. The instrument was operated in data-dependent acquisition (DDA) mode using FT mass analyzer for one survey MS scan on selecting precursor ions followed by Top 3 second data-dependent HCD-MS/MS scans for precursor peptides with 2–7 charged ions above a threshold ion count of 10,000 with normalized collision energy of 37.5%. For these experiments, the MS survey scans were acquired over a mass range of *m/z* 400–1,600 with the Orbitrap analyzer at a resolving power of 120,000 (fwhm at *m/z* 200), and MS/MS scans were acquired at 30,000 resolution with the mass range *m/z* 105–2,000. Dynamic exclusion parameters were set 1 with a 45s exclusion duration with ±10 ppm exclusion mass width. All data were acquired with Xcalibur 3.0 operating software and Orbitrap Fusion Tune 2.0 (Thermo-Fisher Scientific, San Jose, CA).

NanoLC-MRM analyses was performed using a UltiMate3000 nanoLC (Thermo Fisher Scientific, Sunnyvale, CA) coupled with the 4000 QTRAP mass spectrometer (SCIEX, Framingham, MA) [42]. The tryptic peptides from 3 *MSTN*^*−/−*^ and 3 WT samples were individually injected onto an Acclaim PepMap C18 trapping column as described above and then separated on an Acclaim PepMap C18 RP nano column (2 µm, 75 µm × 15 cm) and eluted in a 90 min gradient of 5% to 35% ACN-0.1% FA at 300 nl/min. MRM data acquisition was performed using Analyst 1.6 software (Sciex) after further optimization using MRM-triggered MS/MS analyses [42].

### Protein identification and data analysis

All MS and MS/MS raw spectra of TMT experiments from each set were processed and searched using Sequest HT algorithm within the Proteome Discoverer 1.4 (PD1.4, Thermo). The *Bos Taurus* RefSeq sequence database containing 41,062 entries downloaded from NCBInr database on April 12, 2016 was used for database search. Identified peptides were filtered for maximum 1% FDR using the Percolator algorithm in PD 1.4 along with additional peptide confidence set to high. Only peptide spectra containing all reporter ions were designated as “quantifiable spectra” and used for peptide/protein quantitation. The final lists of protein identification/quantitation were grouped and further filtered by PD 1.4 with at least 2 unique peptides per protein by counting only rank-1 peptides with only in top scored proteins (Supplementary Table 1A). The quantitative protein ratios were weighted and normalized by the median ratio for all quantifiable spectra of the unique peptides pertaining to that protein.

For quantitative phosphopeptides analysis, an additional phosphorylation on Ser, Thr, Tyr residues were specified as variable modifications. To confidently localize phosphorylation sites, the phosphoRS 3.0 node integrated in PD 1.4 workflow was used. The algorithm of phosphoRS 3.0 software enables automated and confident localization of phosphosites by determining individual probability values for each putatively phosphorylated site within the validated peptide sequences [49] (Supplementary Table 2A). For each relative ratio group of phosphopeptides/sites, no normalization was applied.

### Statistical, bioinformatics and motif analysis

To examine the quantitative precision and reproducibility of TMT 6-plex data, we performed careful statistical analyses to assess internal variation in biological triplicates between the two groups as previously described [74]. The internal error plots for assessment of the biological replicate variation were used to establish ratio cutoff. Additionally, the two-sample student *t*-test between the two groups was carried out and *p*-value was obtained for each of the quantified proteins. GO and KEGG analyses were performed by DAVID 6.8 (https://david-d.ncifcrf.gov/), each GO term and pathway with corrected *p*-values ≤ 0.01 were considered to be significantly enriched and reported in this study. Functional interaction networks of the DEPs and DEPPs were predicted using STRING 10.0 (http://string-db.org/) software based on either known or predicted interactions in STRING databases. Motif-x (http://motif-x.med.harvard.edu/) software tool was used for analysis of phosphorylation motif for the identified phosphorylated peptides, all the database protein sequences were used as a background database and other parameters were set to default value in the program.

### MRM analysis and western blot analysis

Multiple Reaction Monitoring (MRM) [75–77], was used for validation of 14 DEPs of most interest, which were selected from the 69 DEPs found in the global proteomics analysis. Skyline 3.1 was used to generate an initial MRM transition pair list for the 14 candidate DEPs as reported previously [78]. Specifically, the PD 1.4 search result file for the TMT 6-plex samples and a database containing the candidate proteins were imported into Skyline 3.1. The initial transition ion list including 3 unique peptides per protein and 3 transition pairs per peptide was exported directly from Skyline 3.1 into an excel file. After mathematical subtraction of the mass of TMT tags in each of the exported peptides, the final list of transition pairs (41 peptides from 14 targeted proteins, see Supplementary Table 3A) for each of the label-free peptides was further confirmed in Skyline using a DDA raw data file for a mixture of 6 unlabeled digests and by a MRM-triggered MS/MS (MRM-IDA) [42]. The resulting MRM raw data were processed using MultiQuant 2.2 software (Sciex, Framingham, MA) (Supplementary Table 3A) as described previously [42, 79]. Western blotting analysis was performed for all 6 experimental samples using antibodies against following 4 proteins: NDUFA6 (1:100, Santa Cruz, Dallas, TX), SUCLG2 (1:100, Santa Cruz, Dallas, TX) p-Akt1 (Ser-473) (1:200, Santa Cruz, Dallas, TX) and reference gene beta-Actin (1:500, Zhongshan Golden Bridge Bio-technology, Beijing, China). Antibody-bound proteins were revealed using ECL for 1 min and visualized with film. Quantification of labelled Western blots was performed using ImageJ analysis software (http://imagej.nih.gov/ij/).

### Measurements of muscle glycogen content and enzyme-linked immuno sorbent assay

Three technical replicates were conducted for each biological replicate gluteus tissue sample. Muscle tissue was rinsed with normal saline, dry with filter paper, and weighed with analytical balance. The muscle sample and lye were added into tube according to the proportion as follow: sample (mg): lye (*µ*l) = 1:3. The tubes were heated in boiling water for 20 minutes and then cooled by running water. Glycogen hydrolysate was further prepared to glycogen assay solution. Muscle glycogen was determined by spectrophotometric analysis (absorbance at 620 nm) according to the method of Muscle Glycogen Content Kit (Nanjing Jiancheng Bioengineering Institute, Nanjing, China).

The protein extracts from the gluteus muscle tissues of *MSTN*^-/-^ and WT beef cattle in three biological replicates were carried out exactly as described above for Enzyme-linked immuno sorbent assays (ELISA). ELISA were performed on PHKA1 (phosphorylase b kinase regulatory subunit alpha, skeletal muscle isoform) and PYGM (glycogen phosphorylase, muscle form) using PHKA1 (Creative Diagnostics, New York, USA) and PYGM ELISA kit (Novus Biologicals, Littleton, USA). Technical triplicates for each of the three biological replicate samples were carried out in each enzyme assay.

## SUPPLEMENTARY MATERIALS FIGURES AND TABLES











## Abbreviations

*MSTN*: *Myostatin*
TGF-β: Transforming Growth Factor-β
MAPK: Mitogen-Activated Protein Kinase
IGF-1: Insulin like Growth Factor 1
*GLUT4*: Glucose Transporter-4
TMT: tandem mass tag
MRM: Multiple Reaction Monitoring
HIV: Human Immunodeficiency Virus
DEPs: Differentially Expressed Proteins
DEPPs: Differentially Expressed Phosphorylated Proteins
PMSF: Phenylmethanesulfonyl Fluoride
BP: Biological Process
CC: Cellular Component
MF: Molecular Function
KEGG: Kyoto Encyclopedia of Genes and Genomes
