# Supplementary material for: Parallel comparative proteomics and phosphoproteomics reveal that cattle myostatin regulates phosphorylation of key enzymes in glycogen metabolism and glycolysis pathway
Source: Oncotarget. 2018 Jan 13;9(13):11352–70. doi: 10.18632/oncotarget.24250 (PMC5834288; doi:10.18632/oncotarget.24250)
Supplement: Supplementary file 1 [file oncotarget-09-11352-s001.pdf]

Parallel comparative proteomics and phosphoproteomics reveal that cattle *Myostatin* regulates phosphorylation of key enzymes in glycogen metabolism and glycolysis pathway

SUPPLEMENTARY MATERIALS

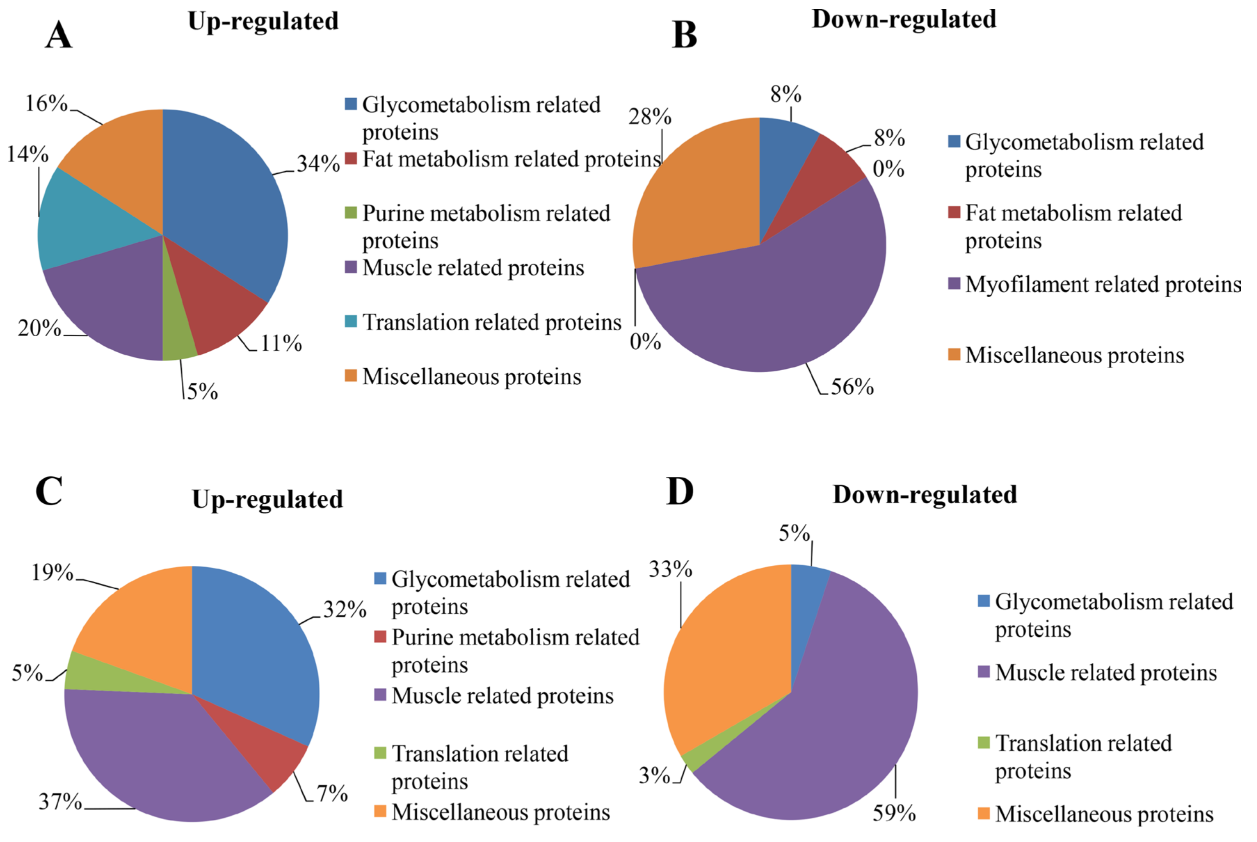

Supplementary Figure 1: The biological functions for 69 differentially expressed proteins and 76 differentially expressed phosphoproteins. (**A**, **B**) show the proportions of biological functions among 44 up-regulated proteins and 25 down-regulated proteins. (**C**, **D**) show the proportions of biological functions among 41 up-regulated phosphoproteins and 39 down-regulated phosphoproteins.

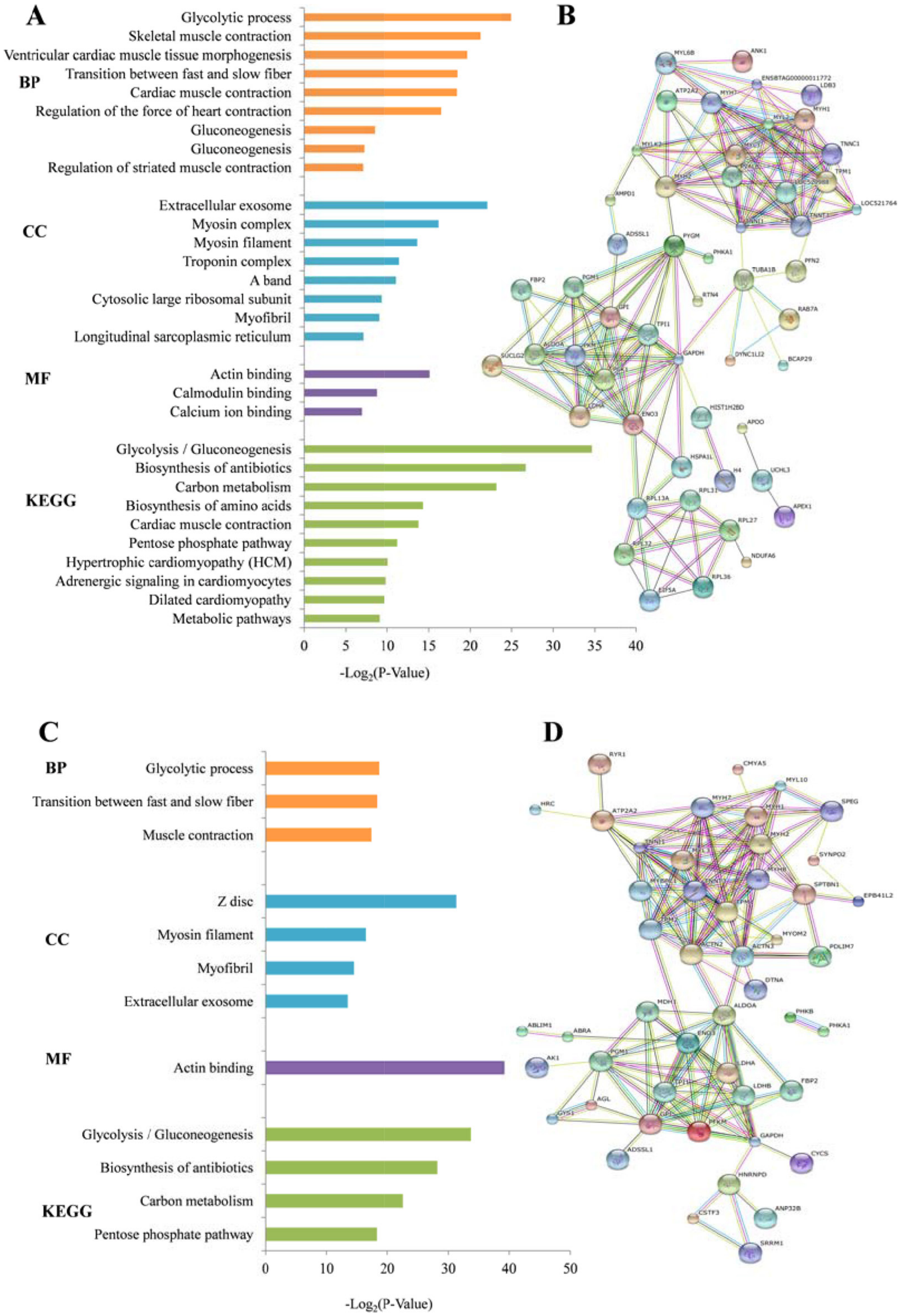

Supplementary Figure 2: Functional classification of differentially expressed proteins and phosphoproteins in *MSTN*$^{-/-}$ vs WT. (**A**, **B**) GO, KEGG and PPI analyses of 69 differentially expressed proteins. The values given in each of enriched terms or pathways are corrected $p \leq 0.01$. (**C**, **D**) GO, KEGG and PPI analyses of 76 differentially expressed phosphoproteins. The values given in each of enriched terms or pathways are corrected $p \leq 0.01$.

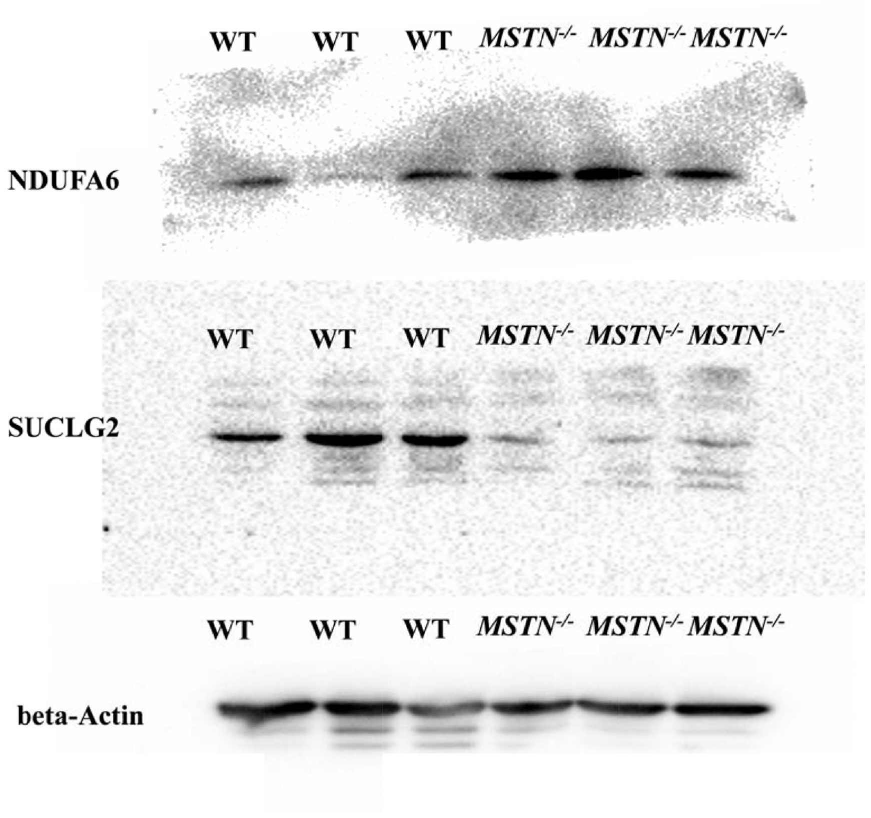

Supplementary Figure 3: Western blot result of NDUFA6 and SUCLG2 proteins.

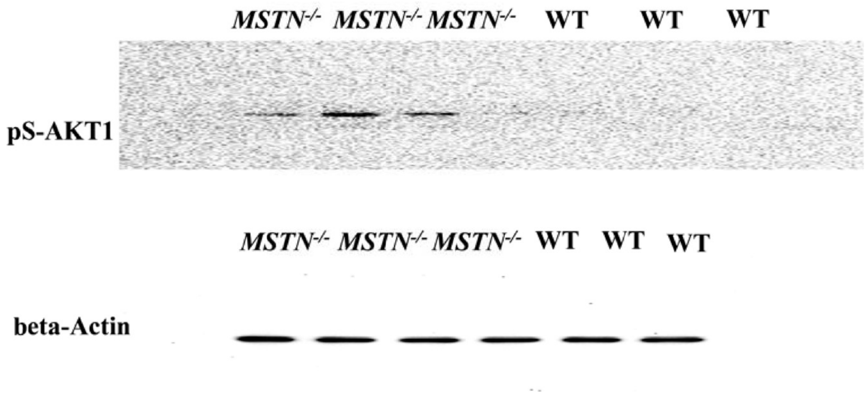

Supplementary Figure 4: Western blot result of pAkt1 (Ser-473).

Supplementary Table 1A: Summary of quantified proteins identified in Luxi beef cattle (*MSTN*$^{-/-}$ vs WT). See Supplementary_Table_1A

Supplementary Table 1B: Summary of the 69 differentially expressed proteins identified in Luxi beef cattle (*MSTN*$^{-/-}$ vs WT). See Supplementary_Table_1B

Supplementary Table 2A: Summary of the quantified phosphoproteins identified in Luxi beef cattle (*MSTN*$^{-/-}$ vs WT). See Supplementary_Table_2A

Supplementary Table 2B: A list of the 149 differentially expressed phosphopeptides corresponding to 76 DEPPs in Luxi beef cattle (*MSTN*$^{-/-}$ vs WT). See Supplementary_Table_2B

Supplementary Table 3A: The result of the 14 differentially expressed proteins selected for MRM-based validation

Accession	Description	L T/C	L sd
41386691	myosin-1 [Bos taurus]	1.526	0.117793399
261245063	myosin-2 [Bos taurus]	0.741	0.051843475
41386711	myosin-7 [Bos taurus]	0.768	0.097533966
139948193	myosin light chain kinase 2, skeletal/cardiac muscle [Bos taurus]	1.412	0.085520383
270483786	myosin light chain 3 [Bos taurus]	0.628	0.079166786
61888856	triosephosphate isomerase [Bos taurus]	1.305	0.08583734
156120479	fructose-bisphosphate aldolase A [Bos taurus]	1.335	0.069818654
77736229	succinyl-CoA ligase [GDP-forming] subunit beta, mitochondrial precursor [Bos taurus]	0.741	0.081195781
114051459	fructose-1,6-bisphosphatase isozyme 2 [Bos taurus]	1.531	0.201063812
115497820	parvalbumin alpha [Bos taurus]	2.165	0.626637
28461265	voltage-dependent L-type calcium channel subunit beta-1 [Bos taurus]	1.351	0.232069013
28461207	NADH dehydrogenase [ubiquinone] 1 alpha subcomplex subunit 6 [Bos taurus]	1.314	0.081335449
70778766	60S ribosomal protein L31 [Bos taurus]	1.371	0.370240682
77404275	60S ribosomal protein L27 [Bos taurus]	1.552	0.24873407
27806559	L-lactate dehydrogenase A chain [Bos taurus]	1.632	0.168804823

Supplementary Table 3B: Summary of the 14 differentially expressed proteins selected for MRM-based validation

Proteinaccession	Protein description	Protein name	Fold-change ± SD (MSTN$^{-/-}$vsWT)	*p*-value	Regulated
41386691	myosin-1	MYH1	1.53 ± 0.12	0.0001	Up
115497820	parvalbumin alpha	PVALB	2.17 ± 0.63	0.0077	Up
28461265	voltage-dependent L-type calcium channel subunit beta-1	CACNB1	1.35 ± 0.23	0.0003	Up
261245063	myosin-2	MYH2	0.72 ± 0.05	0.0027	Down
41386711	myosin-7	MYH7	0.76 ± 0.10	0.0181	Down
270483786	myosin light chain 3	MYL3	0.63 ± 0.80	0.0012	Down
27806559	L-lactate dehydrogenase A chain	LDHA	1.63 ± 0.17	0.0003	Up
156120479	fructose-bisphosphate aldolase A	ALDOA	1.34 ± 0.07	0.0016	Up
61888856	triosephosphate isomerase	TPI1	1.31 ± 0.09	0.0051	Up
114051459	fructose-1,6-bisphosphatase isozyme 2	FBP2	1.53 ± 0.20	0.0003	Up
77736229	succinyl-CoA ligase [GDP-forming] subunit beta	SUCLG2	0.74 ± 0.08	0.0232	Down
28461207	NADH dehydrogenase [ubiquinone] 1 alpha subcomplex subunit 6	NDUFA6	1.31 ± 0.08	6.7E-05	Up
70778766	60S ribosomal protein L31	RPL31	1.37 ± 0.37	0.0495	Up
77404275	60S ribosomal protein L27	RPL27	1.55 ± 0.25	0.0008	Up

Note: Fold change ≥ 1.30 means up-regulated, Fold change ≤ 0.77 means down-regulated, *p*-value <0.05.
