# Supplementary material for: Parallel comparative proteomics and phosphoproteomics reveal that cattle myostatin regulates phosphorylation of key enzymes in glycogen metabolism and glycolysis pathway
Source: Oncotarget. 2018 Jan 13;9(13):11352–70. doi: 10.18632/oncotarget.24250 (PMC5834288; doi:10.18632/oncotarget.24250)
Supplement: Supplementary file 3 [file oncotarget-09-11352-s003.docx]

**Supplemental Table 1b. Summary of the 69 differentially expressed proteins identified in Luxi beef cattle (*MSTN****^-/-^* **vs WT)**

| Protein accession | Protein description | Protein name | Fold-change±SD (*MSTN^-/-^*vsWT) | p-value | Regulated |
| --- | --- | --- | --- | --- | --- |
| **Muscle related proteins** | | | | | |
| 115495599 | myosin-binding protein H | MYBPH | 1.48±0.12 | 0.0022 | Up |
| 741980538 | PREDICTED: troponin T, fast skeletal muscle isoform X32 | TNNT3 | 5.63±2.20 | 0.0300 | Up |
| 41386691 | myosin-1 | MYH1 | 1.53±0.12 | 0.0001 | Up |
| 139948193 | myosin light chain kinase 2, skeletal/cardiac muscle | MYLK2 | 1.41±0.09 | 0.0007 | Up |
| 741894861 | PREDICTED: LOW QUALITY PROTEIN: myosin-binding protein C, fast-type | LOC520988 | 1.71±0.16 | 0.0005 | Up |
| 115497820 | parvalbumin alpha | PVALB | 2.17±0.63 | 0.0077 | Up |
| 28461265 | voltage-dependent L-type calcium channel subunit beta-1 | CACNB1 | 1.35±0.23 | 0.0003 | Up |
| 168804008 | tubulin alpha-1B chain | TUBA1B | 1.44±0.20 | 0.0066 | Up |
| 78369256 | LIM domain-binding protein 3 | LDB3 | 2.03±0.31 | 0.0002 | Up |
| 261245063 | myosin-2 | MYH2 | 0.72±0.05 | 0.0027 | Down |
| 41386711 | myosin-7 | MYH7 | 0.76±0.10 | 0.0181 | Down |
| 741948775 | PREDICTED: myosin-7B isoform X2 | MYH7B | 0.73±0.12 | 0.0126 | Down |
| 115496556 | myosin light chain 6B | MYL6B | 0.70±0.19 | 0.0337 | Down |
| 528988521 | PREDICTED: myosin regulatory light chain 2, ventricular/cardiac muscle isoform isoform X1 | MYL2 | 0.71±0.10 | 0.0083 | Down |
| 270483786 | myosin light chain 3 | MYL3 | 0.63±0.08 | 0.0012 | Down |
| 741954711 | PREDICTED: troponin I, slow skeletal muscle isoform X1 | TNNI1 | 0.69±0.13 | 0.0120 | Down |
| 77735655 | troponin C, slow skeletal and cardiac muscles | TNNC1 | 0.71±0.10 | 0.0071 | Down |
| 58652133 | tropomyosin alpha-3 chain | TPM3 | 0.75±0.16 | 0.0284 | Down |
| 268607682 | heat shock 70 kDa protein 1-like | HSPA1L | 0.75±0.16 | 0.0356 | Down |
| 528988837 | PREDICTED: sarcoplasmic/endoplasmic reticulum calcium ATPase 2 isoform X1 | ATP2A2 | 0.76±0.09 | 0.0106 | Down |
| 115497506 | LIM and cysteine-rich domains protein 1 | LMCD1 | 0.73±0.08 | 0.0011 | Down |
| 78369682 | PDZ and LIM domain protein 1 | PDLIM1 | 0.74±0.18 | 0.0441 | Down |
| 741909981 | PREDICTED: protein phosphatase 1 regulatory subunit 12B isoform X2 | PPP1R12B | 0.75±0.20 | 0.0282 | Down |
| **Energy metabolism related proteins** | | | | | |
| 297493013 | PREDICTED: phosphorylase b kinase regulatory subunit alpha, skeletal muscle isoform isoform X5 | PHKA1 | 1.50±0.22 | 0.0135 | Up |
| 114051459 | fructose-1,6-bisphosphatase isozyme 2 | FBP2 | 1.53±0.20 | 0.0003 | Up |
| 156120479 | fructose-bisphosphate aldolase A | ALDOA | 1.34±0.07 | 0.0016 | Up |
| 116004023 | phosphoglucomutase-1 | PGM1 | 1.48±0.07 | 4.1E-05 | Up |
| 94966765 | glucose-6-phosphate isomerase | GPI | 1.39±0.08 | 0.0010 | Up |
| 77736349 | beta-enolase | ENO3 | 1.31±0.10 | 0.0106 | Up |
| 77735551 | phosphoglycerate kinase 1 | PGK1 | 1.45±0.10 | 0.0015 | Up |
| 77404273 | glyceraldehyde-3-phosphate dehydrogenase | GAPDH | 1.31±0.06 | 0.0011 | Up |
| 61888856 | triosephosphate isomerase | TPI1 | 1.31±0.09 | 0.0051 | Up |
| 28461197 | glycogen phosphorylase, muscle form | PYGM | 1.37±0.23 | 0.0082 | Up |
| 27806559 | L-lactate dehydrogenase A chain | LDHA | 1.63±0.17 | 0.0003 | Up |
| 329664500 | pyruvate kinase | PKM | 1.31±0.13 | 0.0057 | Up |
| 528961976 | PREDICTED: pyruvate kinase PKM isoform X1 | PKM1 | 1.32±0.10 | 0.0027 | Up |
| 115495921 | reticulon-4 isoform 1 | RTN4 | 1.43±0.10 | 0.0467 | Up |
| 28461207 | NADH dehydrogenase [ubiquinone] 1 alpha subcomplex subunit 6 | NDUFA6 | 1.31±0.08 | 6.7E-05 | Up |
| 741914142 | PREDICTED: adiponectin isoform X2 | ADIPOQ | 2.05±0.89 | 0.0238 | Up |
| 167583524 | adipogenesis regulatory factor | ADIRF | 1.49±0.24 | 0.0105 | Up |
| 528995161 | PREDICTED: dehydrogenase/reductase SDR family member 7C isoform X2 | DHRS7C | 1.36±0.15 | 0.0013 | Up |
| 115497246 | MICOS complex subunit MIC26 precursor | APOO | 7.77±6.55 | 0.0353 | Up |
| 300795004 | SH3 and cysteine-rich domain-containing protein 3 | STAC3 | 1.34±0.10 | 0.0004 | Up |
| 77736229 | succinyl-CoA ligase [GDP-forming] subunit beta, mitochondrial precursor | SUCLG2 | 0.74±0.08 | 0.0232 | Down |
| 226693344 | enoyl-CoA delta isomerase 2, mitochondrial | ECI2 | 0.76±0.10 | 0.0120 | Down |
| 77736147 | D-beta-hydroxybutyrate dehydrogenase, mitochondrial precursor | BDH1 | 0.70±0.07 | 0.0022 | Down |
| 77735877 | ES1 protein homolog, mitochondrial | ES1 | 0.77±0.06 | 0.0029 | Down |
| 149773594 | adenylosuccinate synthetase isozyme 1 | ADSSL1 | 1.30±0.15 | 0.0057 | Up |
| 154152079 | AMP deaminase 1 | AMPD1 | 1.38±0.07 | 0.0003 | Up |
| **Translation related proteins** | | | | | |
| 51036244 | eukaryotic translation initiation factor 5A-1 | EIF5A | 1.31±0.15 | 0.0041 | Up |
| 70778766 | 60S ribosomal protein L31 | RPL31 | 1.37±0.37 | 0.0495 | Up |
| 118151344 | 60S ribosomal protein L36 | RPL36 | 1.31±0.08 | 0.0011 | Up |
| 77404275 | 60S ribosomal protein L27 | RPL27 | 1.55±0.25 | 0.0008 | Up |
| 77736513 | 60S ribosomal protein L32 | RPL32 | 2.86±0.18 | 0.0269 | Up |
| 741960152 | PREDICTED: 60S ribosomal protein L13a isoform X1 | RPL13 | 1.38±0.29 | 0.0062 | Up |
| **Miscellaneous proteins** | | | | | |
| 77735683 | protein S100-A2 | S100A2 | 2.71±0.59 | 0.0091 | Up |
| 223278400 | DNA-(apurinic or apyrimidinic site) lyase | APEX1 | 1.33±0.18 | 0.0010 | Up |
| 114053057 | histone H2B type 1-D | HIST1H2BI | 1.44±0.36 | 0.0217 | Up |
| 741971345 | PREDICTED: uncharacterized protein LOC528329 | LOC528329 | 1.32±0.14 | 0.0242 | Up |
| 741969892 | PREDICTED: ras-related protein Rab-7a isoform X1 | RAB7A | 1.94±0.81 | 0.0063 | Up |
| 741977674 | PREDICTED: ankyrin-1 isoform X7 | ANK1 | 1.33±0.13 | 0.0208 | Up |
| 83035093 | proteasome subunit beta type-5 | PSMB5 | 1.38±0.39 | 0.0374 | Up |
| 189491887 | profilin-2 | PFN2 | 0.76±0.09 | 0.0045 | Down |
| 134085803 | protein FAM98A | LOC530070 | 0.67±0.23 | 0.0048 | Down |
| 84000125 | B-cell receptor-associated protein 29 | BCAP29 | 0.67±0.03 | 6.3 E-05 | Down |
| 528992092 | PREDICTED: nucleobindin-1 isoform X2 | NUCB1 | 0.76±0.10 | 0.0086 | Down |
| 82617542 | monocarboxylate transporter 1 | SLC16A1 | 0.71±0.04 | 0.0006 | Down |
| 329663806 | cytoplasmic dynein 1 light intermediate chain 2 | DYNC1LI2 | 0.69±0.07 | 0.0001 | Down |
| 94966875 | ubiquitin carboxyl-terminal hydrolase isozyme L3 | UCHL3 | 0.73±0.08 | 0.0059 | Down |
| 528992092 | PREDICTED: nucleobindin-1 isoform X2 | NUCB1 | 0.76±0.10 | 0.0086 | Down |
| 82617542 | monocarboxylate transporter 1 | SLC16A1 | 0.71±0.04 | 0.0002 | Down |

Note: Fold change ≥1.30 means up-regulated, Fold change ≤0.77 means down-regulated, p-value <0.05
