# Supplementary material for: Parallel comparative proteomics and phosphoproteomics reveal that cattle myostatin regulates phosphorylation of key enzymes in glycogen metabolism and glycolysis pathway
Source: Oncotarget. 2018 Jan 13;9(13):11352–70. doi: 10.18632/oncotarget.24250 (PMC5834288; doi:10.18632/oncotarget.24250)
Supplement: Supplementary file 5 [file oncotarget-09-11352-s005.docx]

**Supplementary Table 2b. A list of the 149 differentially expressed phosphopeptides corresponding to 76 DEPPs in Luxi beef cattle (*MSTN****^-/-^* **vs WT)**

| Protein accession | Protein description | Protein name | Site | Phosphopeptide sequence | Fold-change±SD(*MSTN^-/-^*vsWT) | P-value | Regulated |
| --- | --- | --- | --- | --- | --- | --- | --- |
| **Muscle related proteins** | | | | | | | |
| 41386691 | myosin-1 | MYH1 | s | tLALLFSGPAsGEAEGGPk | 2.03±0.12 | 0.0025 | Up |
| 41386691 | myosin-1 | MYH1 | t | aLEDQLSELktkEDEQQR | 1.59±0.28 | 0.0234 | Up |
| 41386691 | myosin-1 | MYH1 | s | qAEEAEEQSNVNLskFR | 1.56±0.27 | 0.0190 | Up |
| 41386691 | myosin-1 | MYH1 | s | qAEEAEEQSNVNLsk | 1.38±0.10 | 0.0048 | Up |
| 41386691 | myosin-1 | MYH1 | s | tLALLFSGPAsGEAEGGPkk | 1.46±0.12 | 0.0023 | Up |
| 41386691 | myosin-1 | MYH1 | y | qkyEETHAELEASQk | 1.87±0.50 | 0.0197 | Up |
| 41386691 | myosin-1 | MYH1 | s | aLEDQLsELkTk | 1.67±0.40 | 0.0425 | Up |
| 528941306 | PREDICTED: serine/arginine repetitive matrix protein 1 isoform X5 | SRRM1 | s | kPPAPPsPVQsQsPSTNWSPAVPVkk | 1.39±0.16 | 0.0156 | Up |
| 528949469 | PREDICTED: myosin-binding protein C, slow-type isoform X1 | MYBPC1 | s | kDsEWsIGESPAGEEQDk | 2.89±0.42 | 0.0024 | Up |
| 528949469 | PREDICTED: myosin-binding protein C, slow-type isoform X1 | MYBPC1 | s | kDsEWSIGESPAGEEQDkQNANSQLSILFVEkPQGGTVk | 6.81±2.62 | 0.0039 | Up |
| 528949469 | PREDICTED: myosin-binding protein C, slow-type isoform X1 | MYBPC1 | s | kDsEWsIGESPAGEEqDkQNAnSQLSILFVEkPQGGTVk | 4.07±1.60 | 0.0345 | Up |
| 528949469 | PREDICTED: myosin-binding protein C, slow-type isoform X1 | MYBPC1 | s | kDsEWsIGESPAGEEQDkQNANSQLSILFVEkPQGGTVk | 5.38±0.18 | 0.0201 | Up |
| 528949469 | PREDICTED: myosin-binding protein C, slow-type isoform X1 | MYBPC1 | s | kDSEWsIGESPAGEEqDkQNAnSQLSILFVEkPQGGTVk | 8.58±3.96 | 0.0406 | Up |
| 528949469 | PREDICTED: myosin-binding protein C, slow-type isoform X1 | MYBPC1 | s | kDsEWsIGESPAGEEQDkQnANSQLSILFVEkPQGGTVk | 3.16±0.71 | 0.0100 | Up |
| 164420785 | PDZ and LIM domain protein 7 isoform 2 | PDLIM7 | t | vQtPDkQPLRPLVPDASk | 1.92±0.34 | 0.0058 | Up |
| 300794839 | synemin | SYNM | s | aVSESPqEAsAEDmSGNEVTSGVSR | 1.58±0.38 | 0.0476 | Up |
| 114052551 | smoothelin-like protein 2 | SMTNL2 | s | sQsFGVASASSIk | 1.78±0.45 | 0.0397 | Up |
| 115495613 | alpha-actinin-3 | ACTN3 | s | sNIDRLEGDHQLLQEsLVFDNk | 1.52±0.18 | 0.0052 | Up |
| 528976046 | PREDICTED: junctophilin-1 isoform X1 | JPH1 | s | kQNPsSGAR | 1.59±0.30 | 0.0256 | Up |
| 528976046 | PREDICTED: junctophilin-1 isoform X1 | JPH1 | s | aPPkEVGAVVPSSkYsGR | 1.76±0.31 | 0.0155 | Up |
| 528995059 | PREDICTED: myosin-8 isoform X1 | MYH8 | y | qkyEETqTELEASQk | 1.71±0.43 | 0.0281 | Up |
| 62988302 | PDZ and LIM domain protein 7 isoform 1 | PDLIM7 | s | ekYVLELQsPR | 1.50±0.16 | 0.0079 | Up |
| 741916372 | PREDICTED: xin actin-binding repeat-containing protein 2 isoform X2 | XIRP2 | s | gIsAQEIQTGNVk | 2.24±0.19 | 0.0004 | Up |
| 741917677 | PREDICTED: striated muscle preferentially expressed protein kinase isoform X3 | SPEG | s | gLQAAQQVEPTPPSAQDSPsEPk | 1.45±0.17 | 0.0045 | Up |
| 741917677 | PREDICTED: striated muscle preferentially expressed protein kinase isoform X3 | SPEG | s | rSsDTGSk | 1.98±0.39 | 0.0100 | Up |
| 741972301 | PREDICTED: dystrobrevin alpha isoform X10 | DTNA | s | gHAGGSHsNQHQmk | 1.87±0.58 | 0.0469 | Up |
| 741976611 | PREDICTED: actin-binding LIM protein 1 | ABLIM1 | s | sTsQGSINSPVYSR | 1.30±0.11 | 0.0079 | Up |
| 741976990 | PREDICTED: myomesin-2 isoform X1 | MYOM2 | s | nWHEVNsSPLkER | 2.40±0.61 | 0.0068 | Up |
| 741976990 | PREDICTED: myomesin-2 isoform X1 | MYOM2 | s | lLPAAAPsPAH | 1.37±0.23 | 0.0296 | Up |
| 156121271 | sarcoplasmic reticulum histidine-rich calcium-binding protein precursor | HRC | s | dGNGsEEHDRGHGPSHR | 1.65±0.34 | 0.0464 | Up |
| 156121271 | sarcoplasmic reticulum histidine-rich calcium-binding protein precursor | HRC | s | hRNHGEEEEEDDsEEHHHHHSPSHR | 2.01±0.82 | 0.0394 | Up |
| 156121271 | sarcoplasmic reticulum histidine-rich calcium-binding protein precursor | HRC | s | hRnHGEEEEEDDsEEHHHHHSPSHR | 1.44±0.19 | 0.0119 | Up |
| 156121271 | sarcoplasmic reticulum histidine-rich calcium-binding protein precursor | HRC | s | hRDHGEEEDEDDSEEHHHHHGGnkEDEDEDLstEHWHqAPR | 1.46±0.27 | 0.0316 | Up |
| 156121271 | sarcoplasmic reticulum histidine-rich calcium-binding protein precursor | HRC | s | hRGHEEEDDDEDDIVsTEHR | 1.35±0.16 | 0.0175 | Up |
| 156121271 | sarcoplasmic reticulum histidine-rich calcium-binding protein precursor | HRC | s | hRDHGEEEEEDDsEEHHHHHSPSHR | 1.32±0.04 | 0.0003 | Up |
| 156121271 | sarcoplasmic reticulum histidine-rich calcium-binding protein precursor | HRC | s | hRDHGEEEDEDDsEEHHHHHGGnkEDEDEDLSTEHWHQAPR | 1.59±0.46 | 0.0436 | Up |
| 741980510 | PREDICTED: troponin T, fast skeletal muscle isoform X18 | Tnnt3 | y | eLWDTLYQLETDkFEyGEk | 1.50±0.14 | 0.0049 | Up |
| 741980510 | PREDICTED: troponin T, fast skeletal muscle isoform X18 | Tnnt3 | y | akELWDTLYQLETDkFEyGEk | 2.44±0.54 | 0.0080 | Up |
| 139948281 | synaptopodin-2 | SYNPO2 | s | kGPGAGADsGPEEDYLSLGAEAcnFmQGSSAk | 0.60±0.18 | 0.0365 | Down |
| 153791302 | leucine-rich single-pass membrane protein 1 | LSMEM1 | s | rSsQDSGSR | 0.62±0.18 | 0.0408 | Down |
| 156121271 | sarcoplasmic reticulum histidine-rich calcium-binding protein precursor | HRC | s | gHREEEDEDEEEENVsPGYGQQVHR | 0.57±0.11 | 0.0086 | Down |
| 164448592 | four and a half LIM domains protein 1 isoform 1 | FHL1 | s | kPIGADsk | 0.74±0.11 | 0.0459 | Down |
| 261245063 | myosin-2 | MYH2 | s | eQYEEEQEskAELQR | 0.67±0.13 | 0.0325 | Down |
| 261245063 | myosin-2 | MYH2 | s | tLEDQVnELkskEEEQQR | 0.70±0.05 | 0.0015 | Down |
| 261245063 | myosin-2 | MYH2 | t | tLAFLFSGtPTGDSEASGGTk | 0.61±0.09 | 0.0061 | Down |
| 261245063 | myosin-2 | MYH2 |  | ekNDLQLQVQsEAEGLADAEER | 0.58±0.08 | 0.0085 | Down |
| 261245063 | myosin-2 | MYH2 | s | tLEDQVNELkskEEEQQR | 0.48±0.05 | 0.0050 | Down |
| 261245063 | myosin-2 | MYH2 | s | qLDEkEALVSQLsR | 0.62±0.16 | 0.0359 | Down |
| 261245063 | myosin-2 | MYH2 | y | qkyEETHAELEAAQk | 0.49±0.12 | 0.0168 | Down |
| 261245063 | myosin-2 | MYH2 | s | qAEEAEEQSNTNLskFRk | 0.57±0.15 | 0.0406 | Down |
| 261245063 | myosin-2 | MYH2 | t | mEIDDLASNVEtISk | 0.69±0.08 | 0.0024 | Down |
| 261245063 | myosin-2 | MYH2 | s | qAEEAEEQSNTNLskFR | 0.49±0.06 | 0.0022 | Down |
| 270483786 | myosin light chain 3 | MYL3 | s | qEELNsk | 0.58±0.09 | 0.0049 | Down |
| 77736221 | alpha-actinin-2 | ACTN2 | s | qLVPIRDQsLQEELAR | 0.63±0.08 | 0.0074 | Down |
| 300795124 | actin-binding Rho-activating protein | ABRA | s | aPkPSsPkPEGcGEDGRGSEEASAVSPIk | 0.76±0.08 | 0.0133 | Down |
| 331028779 | elongation factor 1-delta isoform 2 | EEF1D | t | kAATATEDDEDDDIDLFGSDEEEDkEAtR | 0.67±0.17 | 0.0443 | Down |
| 41386691 | myosin-1 | MYH1 | t | lINDLTtQRAR | 0.73±0.13 | 0.0490 | Down |
| 41386711 | myosin-7 | MYH7 | s | vkLEQHVDDLEGsLEQEk | 0.53±0.18 | 0.0124 | Down |
| 41386711 | myosin-7 | MYH7 | s | vkLEQHVDDLEGsLEqEkk | 0.51±0.13 | 0.0064 | Down |
| 41386711 | myosin-7 | MYH7 | s | skAEETQRsVNDLTSQR | 0.64±0.14 | 0.0380 | Down |
| 41386711 | myosin-7 | MYH7 | s | iEDEQALGsQLQk | 0.66±0.13 | 0.0140 | Down |
| 41386711 | myosin-7 | MYH7 | s | kmEGDLNEmEIQLsHANR | 0.62±0.08 | 0.0060 | Down |
| 41386711 | myosin-7 | MYH7 | s | nLQEEIsDLTEQLGSSGk | 0.62±0.10 | 0.0054 | Down |
| 41386711 | myosin-7 | MYH7 | s | lEQHVDDLEGsLEQEkk | 0.53±0.10 | 0.0140 | Down |
| 41386711 | myosin-7 | MYH7 | s | kHADsVAELSEQIDNLQR | 0.77±0.04 | 0.0003 | Down |
| 41386711 | myosin-7 | MYH7 | s | aTILsREGGk | 0.50±0.12 | 0.0090 | Down |
| 41386711 | myosin-7 | MYH7 | t | sVNDLtSQR | 0.54±0.05 | 0.0012 | Down |
| 41386711 | myosin-7 | MYH7 | s | lQTENGELsRqLDEkEALISqLTR | 0.60±0.19 | 0.0409 | Down |
| 41386711 | myosin-7 | MYH7 | s | skAEETQRsVnDLTSQR | 0.65±0.08 | 0.0099 | Down |
| 41386711 | myosin-7 | MYH7 | s | tLEDQMNEHRskAEETQR | 0.53±0.19 | 0.0442 | Down |
| 41386711 | myosin-7 | MYH7 | y | gkLTyTQQLEDLkR | 0.54±0.16 | 0.0252 | Down |
| 528925991 | PREDICTED: myosin regulatory light chain 10 isoform X1 | MYL10 | s | aEGGASSnVFSMFDQsQIQEFk | 0.63±0.16 | 0.0326 | Down |
| 528937137 | PREDICTED: probable guanine nucleotide exchange factor MCF2L2 isoform X3 | MCF2L2 | s | iSqPsTLVPHQk | 0.75±0.07 | 0.0055 | Down |
| 528941529 | PREDICTED: myomesin-3 isoform X2 | MYOM3 | s | qTsTVELEER | 0.56±0.14 | 0.0319 | Down |
| 528949469 | PREDICTED: myosin-binding protein C, slow-type isoform X1 | MYBPC1 | t | iLTPLtDQTVNLGk | 0.70±0.09 | 0.0107 | Down |
| 528949469 | PREDICTED: myosin-binding protein C, slow-type isoform X1 | MYBPC1 | s | sAFkRsGEGQDDAGELDFSGLLk | 0.68±0.07 | 0.0113 | Down |
| 528957265 | PREDICTED: synaptopodin isoform X2 | SYNPO | s | sYTTTGQDGLqPTAVsPTYSSDISPVSPSR | 0.68±0.12 | 0.0312 | Down |
| 528957265 | PREDICTED: synaptopodin isoform X2 | SYNPO | s | aAsPAkPSSLDLVPSLPk | 0.77±0.08 | 0.0136 | Down |
| 528958681 | PREDICTED: tropomyosin beta chain isoform X1 | TPM2 | s | aISEELDnALnDITsL | 0.61±0.18 | 0.0328 | Down |
| 528968379 | PREDICTED: spectrin beta chain, non-erythrocytic 1 isoform X2 | SPTBN1 | s | vSEETESqqQWDTSkGEQVsQNGLPAEQGSPR | 0.49±0.12 | 0.0064 | Down |
| 528988837 | PREDICTED: sarcoplasmic/endoplasmic reticulum calcium ATPase 2 isoform X1 | ATP2A2 | t | tVEEVLGHFGVNEStGLSLEQVk | 0.64±0.18 | 0.0442 | Down |
| 528988837 | PREDICTED: sarcoplasmic/endoplasmic reticulum calcium ATPase 2 isoform X1 | ATP2A2 | s | tVEEVLGHFGVNEsTGLSLEQVkk | 0.58±0.12 | 0.0064 | Down |
| 530234515 | cardiomyopathy-associated protein 5 | CMYA5 | s | aSPsPTETAASQYPAWSEVEk | 0.73±0.12 | 0.0480 | Down |
| 58652133 | tropomyosin alpha-3 chain | TPM3 | y | hIAEEADRkyEEVAR | 0.53±0.13 | 0.0123 | Down |
| 741917677 | PREDICTED: striated muscle preferentially expressed protein kinase isoform X3 | SPEG | s | asQEELRSPAGSVAER | 0.70±0.13 | 0.0304 | Down |
| 741917677 | PREDICTED: striated muscle preferentially expressed protein kinase isoform X3 | SPEG | s | rLsQPNLSGSVQEDLGHQYVR | 0.69±0.13 | 0.0326 | Down |
| 58652133 | tropomyosin alpha-3 chain | TPM3 | s | kAADAEAEVAsLNRR | 0.77±0.11 | 0.0396 | Down |
| 741954711 | PREDICTED: troponin I, slow skeletal muscle isoform X1 | TNNI1 | s | kNVEAmsGmEGR | 0.63±0.11 | 0.0363 | Down |
| 741959089 | PREDICTED: ryanodine receptor 1 isoform X4 | RYR1 | t | aAmmTQPPAtPTLPR | 0.67±0.05 | 0.0019 | Down |
| 741968836 | PREDICTED: microtubule-associated protein 4 isoform X4 | MAP4 | s | tNTGRDsPITEmATk | 0.38±0.16 | 0.0426 | Down |
| 741914542 | PREDICTED: TRAF2 and NCK-interacting protein kinase isoform X7 | TNIK | s | sPNsPPISQk | 0.76±0.11 | 0.0325 | Down |
| **Energy related proteins** | | | | | | | |
| 114051459 | fructose-1,6-bisphosphatase isozyme 2 | FBP2 | y | iYSLNEGyAk | 1.52±0.12 | 0.0017 | Up |
| 114051459 | fructose-1,6-bisphosphatase isozyme 2 | FBP2 | y | yFDAATTEYVQk | 1.36±0.19 | 0.0280 | Up |
| 115497288 | ATP-dependent 6-phosphofructokinase, muscle type | PFKM | s | kNVLGHmQQGGsPTPFDR | 1.44±0.16 | 0.0117 | Up |
| 115497288 | ATP-dependent 6-phosphofructokinase, muscle type | PFKM | s | gRsFmnNWEVYk | 1.49±0.09 | 0.0006 | Up |
| 116004023 | phosphoglucomutase-1 | PGM1 | s | fNIsNGGPAPEAITDkIFQISk | 1.63±0.35 | 0.0366 | Up |
| 116004023 | phosphoglucomutase-1 | PGM1 | s | aYQDQkPGTsGLR | 1.58±0.17 | 0.0025 | Up |
| 116004023 | phosphoglucomutase-1 | PGM1 | y | lYIDSyEkDLAk | 1.50±0.15 | 0.0021 | Up |
| 155372313 | glycogen [starch] synthase, muscle | GYS1 | s | yPRPAsVPPsPSLSR | 1.44±0.17 | 0.0066 | Up |
| 156120479 | fructose-bisphosphate aldolase A | ALDOA | s | gILAADEsTGSIAk | 1.47±0.16 | 0.0074 | Up |
| 156120479 | fructose-bisphosphate aldolase A | ALDOA | t | tVPPAVPGItFLSGGQSEEEASINLNAINk | 2.08±0.14 | 0.0028 | Up |
| 156120479 | fructose-bisphosphate aldolase A | ALDOA | s | rTVPPAVPGITFLsGGQSEEEASINLNAINk | 1.64±0.28 | 0.0073 | Up |
| 156120479 | fructose-bisphosphate aldolase A | ALDOA | s | vDkGVVPLAGTnGETTTQGLDGLsER | 1.42±0.30 | 0.0473 | Up |
| 156120479 | fructose-bisphosphate aldolase A | ALDOA | t | tVPPAVPGItFLSGGQSEEEASINLnAINk | 1.50±0.32 | 0.0017 | Up |
| 156120479 | fructose-bisphosphate aldolase A | ALDOA | t | lQSIGTENtEENRR | 1.33±0.15 | 0.0196 | Up |
| 156120479 | fructose-bisphosphate aldolase A | ALDOA | s | tVPPAVPGITFLsGGQSEEEASInLnAINk | 1.89±0.39 | 0.0025 | Up |
| 27806559 | L-lactate dehydrogenase A chain | LDHA | y | qVVDSAyEVIk | 1.44±0.14 | 0.0032 | Up |
| 300794727 | glycogen debranching enzyme | AGL | t | yTWtDVGQLVQk | 1.36±0.14 | 0.0108 | Up |
| 358416561 | PREDICTED: LOW QUALITY PROTEIN: phosphorylase b kinase regulatory subunit beta | PHKB | t | rQSStSnAPEQEQkPDVTMTEWR | 1.34±0.09 | 0.0028 | Up |
| 529000048 | PREDICTED: adenylosuccinate synthetase isozyme 1 isoform X4 | ADSSL1 | s | aSNDRPPsAGGVkR | 1.90±0.40 | 0.0016 | Up |
| 529000048 | PREDICTED: adenylosuccinate synthetase isozyme 1 isoform X4 | ADSSL1 | s | aSnDRPPsAGGVkR | 2.27±0.24 | 0.0005 | Up |
| 529014933 | PREDICTED: phosphorylase b kinase regulatory subunit alpha, skeletal muscle isoform isoform X6 | PHKA1 | s | rLsISTESQVk | 2.16±0.42 | 0.0097 | Up |
| 61888850 | adenylate kinase isoenzyme 1 | AK1 | s | aEVsSGSAR | 1.38±0.09 | 0.0012 | Up |
| 61888856 | triosephosphate isomerase | TPI1 | s | eLAsQPDVDGFLVGGASLkPEFVDIINAk | 1.61±0.39 | 0.0378 | Up |
| 61888856 | triosephosphate isomerase | TPI1 | s | sNVSDAVAQsAR | 1.30±0.16 | 0.0422 | Up |
| 61888856 | triosephosphate isomerase | TPI1 | s | hVFGEsDELIGQk | 1.40±0.21 | 0.0230 | Up |
| 77404273 | glyceraldehyde-3-phosphate dehydrogenase | GAPDH | t | vIHDHFGIVEGLMTTVHAITAtQk | 1.90±0.25 | 0.0285 | Up |
| 77404273 | glyceraldehyde-3-phosphate dehydrogenase | GAPDH | t | vIHDHFGIVEGLmTTVHAITAtQk | 1.57±0.49 | 0.0167 | Up |
| 77404273 | glyceraldehyde-3-phosphate dehydrogenase | GAPDH | s | gAAQNIIPAsTGAAk | 1.59±0.24 | 0.0076 | Up |
| 77404273 | glyceraldehyde-3-phosphate dehydrogenase | GAPDH | t | aItIFQERDPANIk | 1.64±0.52 | 0.0427 | Up |
| 77736349 | beta-enolase | ENO3 | s | eILDsRGNPTVEVDLHTAk | 1.47±0.20 | 0.0104 | Up |
| 94966765 | glucose-6-phosphate isomerase | GPI | t | iEPELDGSSPVtSHDSSTNGLINFIk | 1.71±0.32 | 0.0288 | Up |
| 94966765 | glucose-6-phosphate isomerase | GPI | s | kIEPELDGSsPVTSHDSSTnGLINFIk | 1.43±0.26 | 0.0106 | Up |
| 94966765 | glucose-6-phosphate isomerase | GPI | s | kIEPELDGsSPVTSHDSSTnGLInFIk | 2.05±0.19 | 0.0004 | Up |
| 94966765 | glucose-6-phosphate isomerase | GPI | s | kIEPELDGSsPVTSHDSSTNGLINFIk | 1.56±0.16 | 0.0272 | Up |
| 27806561 | L-lactate dehydrogenase B chain | LDHB | s | iVADkDYsVTANSk | 0.64±0.16 | 0.0292 | Down |
| 77736203 | malate dehydrogenase, cytoplasmic | MDH1 | s | nVIIWGNHSsTQYPDVNHAk | 0.67±0.11 | 0.0013 | Down |
| **Miscellaneous proteins** | | | | | | | |
| 78045555 | acidic leucine-rich nuclear phosphoprotein 32 family member B | ANP32B | t | kREtDDEGEDD | 1.31±0.06 | 0.0009 | Up |
| 157427926 | inactive rhomboid protein 1 | RHBDF1 | s | rDSssSLQHk | 1.60±0.27 | 0.0112 | Up |
| 528949505 | PREDICTED: nascent polypeptide-associated complex subunit alpha isoform X2 | NACA | s | rAsATPNTk | 1.90±0.51 | 0.0300 | Up |
| 528973617 | PREDICTED: RNA-binding protein 39 isoform X3 | RBM39 | s | dksPVREPIDNLTPEER | 1.56±0.24 | 0.0230 | Up |
| 741920357 | PREDICTED: AMP deaminase 1-like | LOC104971550 | s | tISLsVPQTETSSTk | 1.62±0.18 | 0.0028 | Up |
| 741938103 | PREDICTED: band 4.1-like protein 2 isoform X12 | EPB41L2 | s | eVAENqQNQTSDPEEEkGsQSSPPAESQSSPRR | 1.56±0.32 | 0.0413 | Up |
| 741979959 | PREDICTED: neuroblast differentiation-associated protein AHNAK isoform X5 | AHNAK | s | aGVAGSPEASVSGskGDLk | 1.37±0.12 | 0.0076 | Up |
| 136255546 | coatomer subunit beta' | COPB2 | s | sAAQQELDGkPAsPTPVIVTSqTANkEEk | 1.64±0.32 | 0.0157 | Up |
| 148230364 | heterogeneous nuclear ribonucleoprotein D0 | HNRPD | s | nEEDEGHSnsSPR | 2.77±1.58 | 0.0207 | Up |
| 741973682 | PREDICTED: protein FAM195A isoform X1 | FAM195A | t | rPPAtSPSLEGTQEPYTLAHEENVR | 0.59±0.13 | 0.0103 | Down |
| 77735829 | carbonic anhydrase 3 | CA3 | s | qFHLHWGsSDDHGSEHSVDGVk | 0.50±0.19 | 0.0366 | Down |
| 77735829 | carbonic anhydrase 3 | CA3 | t | eISHDPSLkPWtASYDPGSAk | 0.45±0.11 | 0.0135 | Down |
| 77735829 | carbonic anhydrase 3 | CA3 | s | kLsSAMSAAk | 0.67±0.09 | 0.0156 | Down |
| 78045541 | palmdelphin | PALMD | s | qsSSPPcQEDEkDIR | 0.68±0.06 | 0.0032 | Down |
| 82617542 | monocarboxylate transporter 1 | SLC16A1 | s | skGsLQEAGkYETk | 0.62±0.07 | 0.0026 | Down |
| 114051487 | cytochrome c | CYC | s | kTGQAPGFsYTDANk | 0.74±0.10 | 0.0408 | Down |
| 528947704 | PREDICTED: protein FAM115A isoform X2 | TCAF1 | y | dSLGVycIDAYnETMtEkLVk | 0.71±0.11 | 0.0266 | Down |
| 528954959 | PREDICTED: UV excision repair protein RAD23 homolog A isoform X1 | RAD23A | t | aVEYLLtGIPGSPEPEHGSVQESqVSEqPSTEAAGENPLEFLR | 0.68±0.07 | 0.0058 | Down |
| 741890835 | PREDICTED: cleavage stimulation factor subunit 3 isoform X1 | CSTF3 | s | rPNEDsDEDEEkGAVVPPVHDIYR | 0.74±0.12 | 0.0360 | Down |
| 741897747 | PREDICTED: dynein intermediate chain 2, axonemal-like, partial | LOC783827 | s | lAVAyscLnFQR | 0.76±0.07 | 0.0079 | Down |
| 528992246 | PREDICTED: vasodilator-stimulated phosphoprotein isoform X4 | VASP | s | kVskQEEASAGPVAPk | 0.61±0.07 | 0.0017 | Down |
| 529016080 | PREDICTED: matrix-remodeling-associated protein 5 isoform X2 | MXRA5 | t | vDcLAtGLPNPEISWSLPDGSLVNsFmQADDsGGR | 0.41±0.06 | 0.0004 | Down |

Note: Fold change ≥1.30 means up-regulated, Fold change ≤0.77 means down-regulated, p-value <0.05.

The four phosphoproteins with both up-regulated and down-regulated phosphopeptides were highlighted by a yellow.
